# Establishment of a research policy for supportive and palliative care in Japan

**DOI:** 10.1093/jjco/hyab008

**Published:** 2021-02-10

**Authors:** Sadamoto Zenda, Yosuke Uchitomi, Tatsuya Morita, Takuhiro Yamaguchi, Akira Inoue

**Affiliations:** Department of Radiation Oncology, National Cancer Center Hospital East, Chiba, Japan; Innovation Center for Supportive-Palliative and Psychosocial Care, National Cancer Center Hospital, Tokyo, Japan; Innovation Center for Supportive-Palliative and Psychosocial Care, National Cancer Center Hospital, Tokyo, Japan; Palliative and Supportive Care Division, Seirei Mikatahara General Hospital, Shizuoka, Japan; Division of Biostatistics, Tohoku University Graduate School of Medicine, Miyagi, Japan; Department of Palliative Medicine, University Graduate School of Medicine, Miyagi, Japan

**Keywords:** supportive care, palliative care, clinical research, clinical study

## Abstract

**Background:**

While several small groups in Japan have attempted to conduct prospective studies in the field of supportive and palliative care, development of exploratory research into multicentre confirmatory studies has been difficult. The main reason for this is the difference in clinical research methodology in supportive and palliative care compared with medical oncology in terms of the style of multidisciplinary approaches, study design and endpoints. Here, we establish a new research policy for cancer supportive and palliative care in Japan.

**Methods:**

The first draft was developed by a policy working group within the Japanese Supportive, Palliative and Psychosocial Care Study Group. A provisional draft was subsequently developed after review by nine Japanese scientific societies (Japanese Association of Supportive Care in Cancer, Japanese Society of Medical Oncology, Japanese Society of Clinical Oncology, Japanese Society of Palliative Medicine, Japanese Society of Cancer Nursing, Japanese Society of Pharmaceutical Oncology (JASPO), Japan Cancer Association (JCA), Japanese Society of Therapeutic Radiation Oncology and Japanese Cancer Association) and receipt of public comments. The final research policy in the area of supportive and palliative care in Japan (Ver1.0) was completed in December 2018 and underwent its first revision (Ver1.1) in February, 2020.

**Results:**

The policy includes the following components of clinical research: (i) objective of the research policy in the areas of supportive and palliative care; (ii) definitions of supportive care and palliative care; (iii) characteristics of supportive and palliative care research; (iv) target population for research; (v) research design; (vi) endpoints and assessment measures; (vii) handling of the deaths of subjects and (viii) operational structure and quality management.

**Conclusions:**

We hope that studies conducted according to this policy will play important roles in the future development of the supportive and palliative field.

## Teaser

A new research policy for supportive and palliative care was established. Studies conducted according to this policy will play important roles in the future development of these fields.

## Introduction

According to the Cancer Registry and Statistics, Cancer Information Service, National Cancer Center, Japan, ~1.1 million new patients with cancer are recorded in Japan every year (Ministry of Health, Labour and Welfare, National Cancer Registry). Almost all patients receive some kind of cancer treatment, such as surgery, radiotherapy and/or chemotherapy, and experience some form of toxicity induced by the cancer treatment. It is therefore paramount that patients receive supportive care for toxicities based on high-quality evidence.

Patients who receive palliative care should receive evidence-based medical care to enable them to live a comfortable daily life. The clinical research system in Japan, especially that for supportive and palliative care, is plagued by serious problems. Owing to these problems, several small groups in Japan that have attempted to conduct prospective studies in the supportive and palliative care field have found it difficult to develop their exploratory research into multicentre confirmatory studies.

Issues related to clinical research in the supportive and palliative care field can be divided into two major categories: scientific and structural. One scientific problem is the existence of differences in the clinical research methodology in supportive and palliative care compared with medical oncology.

In medical oncology research field, survival-related index (overall survival, progression free survival, etc.) is selected as the primary endpoint in most studies, because the primary aim of these studies is ‘cure’ or ‘prolong survival’. On the other hand, in the supportive and palliative care field, the primary aim is not survival index, but quality of life for long survivors or other index that can be clarified with short term (<1–3 month) because of short prognosis in patient receiving palliative care. There are many non-validated outcome measure in supportive and palliative care field and this problem also makes the study results confusing (1).

More, while the patient characteristics of medical oncology researches except early phase trials are unified, patients with various cancer are included in the same study in supportive and palliative care field (e.g. the study of medication for nausea and vomiting (2).

**Figure 1.**
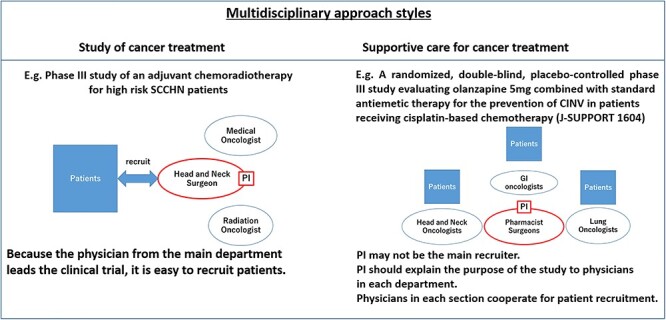
(Left) Clinical trial of a cancer treatment. The physician from the main department leads the clinical trial, making it is easy to recruit patients. (Right) Clinical trial in supportive and palliative care. The principle investigator (PI) may not be the main recruiter. The PI should explain the purpose of the study to physicians in each department. It is important that physicians understand the study and have the same persuasive knowledge-based enthusiasm for the study as the PI for recruitment.

In terms of structural problems, one example is that the working style of co-medical staff differs from that of physicians. Co-medical staff may not have daily shifts. In this case, principle investigators who are nurses or other co-medical staff may have to ask physicians or non-core member co-medical staff to enrol patients on their behalf. However, it is often difficult to enrol patients if the recruiter lacks persuasive knowledge-based enthusiasm when speaking about a clinical trial. This forms one of the most important structural problems in this field (Fig. 1).

In Japan, co-medical staff, including nurses, have little training in clinical trials, although they have often good ideas which can change practice.

We propose that to ensure high-quality studies, it is necessary to establish rules governing research in the area of supportive and palliative care. Here, we report the establishment of a new research policy of cancer supportive and palliative care in Japan.

The main scope of this project was the distinction the methodology of clinical research of supportive and palliative care for cancer patients from that of general oncology. So, this policy is adapted only to clinical trials about oncology, although palliative care and supportive care are performed for all patients with severe diseases.

## Materials and methods

### Aim

The objective of this policy is to produce a set of guidelines for the implementation of clinical research, particularly clinical studies, in supportive care and palliative care. Given that different specialty areas have different ideals regarding supportive and palliative care research, this policy provides a basic common framework that can be shared across different specialty areas. This policy primarily focuses on pharmacotherapies and medical interventions but may be used as a reference for planning clinical studies on non-invasive or minimally invasive interventions, including certain psycho-behavioural interventions, nursing interventions or rehabilitation. This document aims to establish the basic policy for clinical research in supportive and palliative care, and thus does not apply to clinical research/studies/trials regulated by the Clinical Trials Act, i.e. clinical research/studies/trials of unapproved or off-label drugs/devices conducted under the Pharmaceuticals, Medical Devices and Other Therapeutic Products Act and funded by drug/device companies.

Development of the research policy was started by the policy working group within the Japanese Supportive, Palliative and Psychosocial Care Study Group (J-SUPPORT).

### Japanese Supportive, Palliative and Psychosocial Care Study Group

J-SUPPORT was established as an opened hub of a multi-institutional collaborative clinical research for supportive, palliative and psychosocial care on February 2016 and started research managements throughout Japan. (https://www.j-support.org/).

J-SUPPORT conference consists of two type meeting; weekly meeting with core member and monthly meeting with operating officers. We cooperate in providing consultation services and expert advice on clinical research design and statistical analysis to investigators as they launch new research projects in the field of supportive, palliative and psychosocial care.

### First draft

The policy working group had started from November 2016. The policy working group includes the following experts: a palliative care physician, medical oncologist, radiation oncologist, psycho-oncologist and statistician. The backbone of the policy was developed over daily discussions through e-mail and weekly J-SUPPORT conference. First, we discussed the components and detailed items of the research policy. Policy working group members subsequently identified relevant items based on their expertise. A first draft was completed in January 2018 (Ver0.1).

### Provisional draft

The first draft was critically reviewed by the following medical societies: the Japanese Society of Clinical Oncology, Japanese Society of Medical Oncology, Japanese Society of Cancer Nursing, Japanese Society of Palliative Medicine, Japanese Society of Therapeutic Radiation Oncology, Japanese Association of Supportive Care in Cancer and Japan Psycho-Oncology Society. Based on requests from these groups, the J-SUPPORT policy working group modified the first draft to establish a provisional draft in August 2018.

The provisional draft was subsequently subjected to review by the public. The J-SUPPORT policy working group modified the first draft according to the public’s comments to establish the first edition (Ver1.0) in November 2018.

The first edition was modified by incorporating comments from an additional review by the Japanese Cancer Association and Japanese Society of Pharmaceutical Oncology to produce the current edition, Ver1.1 (February 2020).

## Results

This section briefly describes the contents of The Research Policy in the Area of Supportive and Palliative Care in Japan.

This policy includes the following components of clinical research: (i) objective of the research policy in the areas of supportive and palliative care; (ii) definitions of supportive care and palliative care; (iii) characteristics of supportive and palliative care research; (iv) target population for research; (v) research design; (vi) endpoints and assessment measures; (vii) handling of the deaths of subjects and (viii) operational structure and quality management. Amongst these the most important section is ‘Definitions of supportive care and palliative care’.

### Definitions of supportive care and palliative care

The terms ‘supportive care’ and ‘palliative care’ in their broad sense overlap and cannot be differentiated (3–5). However, for the purposes of promoting clinical research (studies), this policy would benefit from narrower definitions of the terms to enable clarification of their core meanings, and thereby the services these terms are meant to incorporate. Thus, in this policy document, the original meaning of ‘supportive care’ (i.e. prevention or relief of complications associated with cancer treatment) is more appropriate; therefore, ‘Supportive care’ is defined as ‘supportive care for side effects induced by cancer treatment’. On the other hand, amongst the palliative care services, those involving pharmacotherapy or invasive treatment are sometimes referred to as ‘palliative medicine’. Given that this policy document focuses on research studies involving medical interventions such as pharmacotherapies, the term ‘palliative care’ is defined as ‘palliative care for cancer-induced symptoms’. These definitions do not negate the existence of other terms currently used to refer to general clinical concepts.

#### Definition of cancer treatment

Cancer treatment refers to treatment that directly acts on a tumour to exert antitumour effects (disease-modifying effects) and specifically includes surgical treatment, cancer pharmacotherapy and radiotherapy.

#### Definition of supportive care (supportive care for toxicities arising from cancer treatment)

The term ‘supportive care’ is interpreted in Japanese as ‘shijiryoho’. Supportive care refers to treatment performed for the prevention or symptomatic relief of adverse reactions to cancer treatment. Adverse reactions include post-treatment complications and sequelae.

#### Definition of palliative care (palliative care for symptoms arising from cancer)

The term ‘palliative care’ is interpreted in Japanese as ‘kanwachiryo’. Palliative care refers to treatment performed for the prevention or symptomatic relief of cancer-related pain, discomfort or symptoms.

### Characteristics of supportive and palliative care research

The main subject is ‘What is different from that of general oncology?’ (6–8).

#### Relationship between cancer treatment and supportive care

Given that supportive care refers to treatment for adverse reactions that occur as a result of cancer treatment, any study protocol for a novel supportive care strategy is prepared on the assumption that the target cancer treatment would be widely used in clinical practice in the future. If the target cancer treatment is not used in clinical practice, its associated supportive care will not be used. Thus, such a study plan should be carefully prepared.

In planning a clinical study, the research group should include researchers from the department that will be providing the treatment, and a cooperative relationship should be established so that co-researchers from the department providing cancer treatment will also play a central role in the study. This is important because such cooperation has a great influence on all steps in the clinical study, including the development speed, patient recruitment and dissemination and implementation of research results.

#### Characteristics of palliative care research

In palliative care research, flexibility of protocol treatment is necessary compared with general oncology.

Characteristically, palliative care in clinical practice is often modified according to the patient’s condition because the patient’s response to a given palliative care quickly becomes apparent (i.e. in several hours to several days).

This means that, to reflect treatments in clinical practice, the criteria for dose reduction, delay and discontinuation of protocol treatment should be flexible. For example, the dose of analgesic medication should be allowed to be titrated to achieve the necessary analgesia according to the patient’s condition.

### Target population for research

In research studies in supportive care and palliative care, consideration of the items below is recommended to facilitate the dissemination and implementation of the study results in clinical practice.

#### Target population for supportive care research

The study results must be adopted in clinical practice as promptly and as broadly as possible. Therefore, to allow for better extrapolability of the study results to the actual patient population in clinical practice, even those with minor differences in cancer treatment (e.g. drugs, procedures), eligibility criteria should not be too restrictive.

Navari et al. (2) reported phase 3 study about triplet combination with or without olanzapine as an anti-emetic treatment and, in this study, patients who were scheduled to receive highly emetogenic chemotherapy (HEC) (either cisplatin at a dose ≥70 mg per square metre of body-surface area, with or without other chemotherapeutic agents, or doxorubicin at a dose of 60 mg per square metre plus cyclophosphamide at a dose of 600 mg per square metre) were recruited. Also in the Japanese large-scale phase 3 study about anti-emetic treatment (J-FORCE study) (9), patients with a malignant solid tumour who were scheduled to be treated with first-line cisplatin (≥50 mg/m^2^) were included. Both the studies were conducted with consideration for implementation to clinical practice. Researchers should keep in mind that the ultimate goal of clinical studies in supportive care is ‘wider dissemination and better implementation of the obtained findings in clinical practice’.

#### Target population for palliative care research

In research in palliative care, the diagnostic criteria for the study population may not be clear. Wherever possible, however, the study population should be defined using diagnostic criteria with an international consensus. For example, the diagnostic criteria for neuropathic pain may be based on the definition provided by the International Association for the Study of Pain (10), whereas the diagnostic criteria for depression may be based on the criteria in the Diagnostic and Statistical Manual of Mental Disorders (11).

#### Definition of advanced cancer patients

To define a patient population in palliative care settings, it is often necessary to define patients with advanced cancer. Previously, the terms ‘terminally ill cancer patients’ and ‘incurable cancer patients’ were used (12,13). However, these terms have unclear definitions and are not recommended. In most studies, the study population can be described using the definition ‘metastatic or locally advanced cancer patients’. Cancer types may be specified, such as ‘clinical stage IV lung cancer’.

### Research design

The main subject is ‘What is the difference between supportive care for side effects and palliative care for symptom from cancer itself in study design’.

This section provides an outline of the research designs used in clinical studies in supportive care or palliative care. Detailed procedures for research methods are provided in separate sections. Although some important points regarding research design apply to both supportive care and palliative care, this section describes points with specific importance to supportive care and palliative care under separate headings.

### Endpoints and assessment measures

This section describes concepts related to endpoints in the planning of studies in supportive care and palliative care. Although there are important points that apply to both supportive care and palliative care, this section describes the points for supportive care and palliative care under separate headings.

#### Supportive care

**Figure 2.**
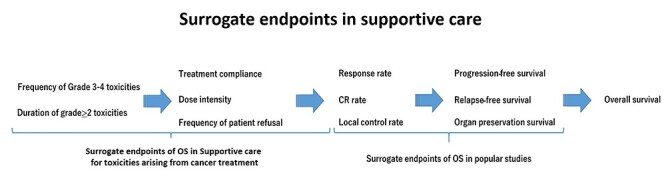
In palliative care, overall survival is not the final goal, and the objectivity of endpoints is one of the most important problems. Supportive care for toxicities from cancer treatment contributes indirectly to improving overall survival. Therefore, it is important to select appropriate surrogate endpoints that reflect the efficacy of supportive care for toxicities from cancer treatment. OS, overall survival; CR, complete response.

Given that supportive care aims to prevent or reduce the occurrence of adverse events in cancer treatment, it should ideally maximize the potential of cancer treatment and improve the treatment results (14). From this perspective, the primary endpoint may be survival-related outcomes such as overall survival. In reality, however, cancer treatment is the main factor affecting the treatment results, and thus, outcomes such as overall survival and relapse-free survival may be inappropriate efficacy endpoints for supportive care (Fig. 2).

Zenda et al. (15) reported the results of multicentre phase 2 study of an opioid-based pain control program for head and neck cancer patients receiving chemoradiotherapy. In this study, cancer treatment completion rate was selected as the primary endpoint.

The results show that patients who completed radiotherapy within 6 weeks have significantly longer survival than patients who did not complete radiotherapy.

However, acute adverse events are speculated to be responsible for half of the patients not completing radiotherapy.

Their hypothesis is that the reduction of the incidence of acute adverse events may reduce treatment interruption and the reduction of treatment interruption may contribute to better treatment outcome.

#### Palliative care

In clinical trials in palliative care that aim to reduce patients’ symptoms, the endpoint should be patients’ symptoms (16–19). Examples of endpoints include severity of pain for analgesic medications and severity of nausea/vomiting for antiemetic medications.

The most appropriate measure of symptoms is often unclear. For example, endpoints for pain can include the worst pain or average pain in 24 h (16). Endpoints for nausea/vomiting can include the worst nausea in 24 h and number of vomiting episodes. As described above, even when the symptoms being treated by palliative care are clear, multiple methods are often available for their assessment. Although determination of the most important endpoints for specific types of symptoms is needed in the future, there are currently no international standardized endpoints for a large number of symptoms.

Thus, it is currently reasonable to employ a primary endpoint that is considered most important for patients and secondary endpoints that are considered relatively less important, with careful reference to prior studies. When multiple endpoints are similarly important, they should be selected as co-primary endpoints (17).

### Handling of the deaths of subjects

Appropriate assessment of serious adverse events (SAEs), which can occur frequently during clinical studies, especially in palliative care, is important for both the safety and efficiency of studies (20,21). On the premise that the occurrence of SAEs is rare, any SAE at a study site should be promptly reported in detail to the ethics committee and independent data monitoring committee.

However, in clinical studies in supportive care or palliative care, particularly palliative care, the occurrence of many SAEs (including deaths) is expected because a subject’s condition can worsen due to worsening of the primary disease during the course of a study. Frequent reporting and assessment of SAEs regarding deaths due to clinical deterioration that are clearly unrelated to any study treatment can therefore not only affect the conduct of the clinical study, but may also lead to the overlooking of truly important SAEs.

When a protocol stipulates that deaths due to deterioration of the primary disease that are unrelated to any study treatment do not require expedited reporting, the following requirements must be fulfilled by the responsible researcher at the study site:

- Assessment of whether the death was expected
- Confirmation that the death was due to deterioration of the primary disease and was unrelated to any study treatment
- Confirmation using clinical documents such as medical charts

### Operational structure and quality management

#### Basic principals

Target quality levels differ across studies, including clinical studies in supportive care and palliative care (22,23). Study costs, time and feasibility should be considered according to applicable regulatory requirements and the research design. In addition to the protection of subjects and quality assurance, efficiency and cost-effectiveness should also be considered, and a research quality management system (including an operational structure) should be established. Specifically, ideal personnel for this role include the research operations office and clinical research support organizations of a data centre or a data manager, biostatistician and clinical research associate (as needed). Given that research methods can differ depending on the research organization, a flexible and feasible system is needed.

In addition, rules regarding authorship, which should reflect the degree of contribution of those involved in the planning and conduct of the study, should be established with a consensus amongst research collaborators before the start of the study because supportive/palliative care studies typically involve multiple departments, specialists and study sites.

## Discussion

The clinical research methodology used in supportive and palliative care differs from that in medical oncology in terms of the style of multidisciplinary approaches, study design and endpoints. Therefore, it is necessary to establish a guideline containing both general remarks and detailed expositions to ensure that researchers have sufficient information to conduct research in the supportive and palliative care field. This guideline defines the basic concepts of supportive and palliative care research with consideration for the minor differences between the specific fields. After finalizing the policy in Japan, we will seek for it to be recognized as a global policy by the Multinational Association of Supportive Care in Cancer through the Japan Cancer Supportive Care Society.

Based on this policy, we have initiated a new mission called the ‘Detailed Exposition-making Project’. Additionally, each core working group within J-SUPPORT is developing a rule book related to toxicities and symptoms (chemotherapy-induced nausea and vomiting, dyspnea, mucositis and pain, etc.) for clinical trials.

We hope that this policy will enable the conduct of high-quality clinical studies and the reporting of reliable evidence in the supportive and palliative care field.

## Supplementary Material

J-SUPPORT-ver1_1_hyab008

20200206_ver1_1_hyab008
