# Supplementary material for: Establishment of a research policy for supportive and palliative care in Japan
Source: Jpn J Clin Oncol. 2021 Feb 10;51(4):538–43. doi: 10.1093/jjco/hyab008 (PMC8129624; doi:10.1093/jjco/hyab008)
Supplement: J-SUPPORT-ver1_1_hyab008 [file j-support-ver1_1_hyab008.pdf]

国立研究開発法人日本医療研究開発機構（AMED）
革新的がん医療実用化研究事業　課題番号 18ck0106376h0002

支持／緩和治療領域研究の方法論確立に関する研究

支持療法・緩和治療領域研究ポリシー

（総論）

Ver1.0　2018 年 12 月 3 日　発行

Ver1.1　2019 年　11 月 8 日　発行

作成：

支持療法・緩和治療研究ポリシー総論作成 WG

国立研究開発法人国立がん研究センター東病院　放射線治療科　医長

全田　貞幹（支持療法）

聖隷三方原病院　副院長／緩和支持治療科　部長
森田　達也（緩和治療）

東北大学大学院医学系研究科緩和医療学分野　教授
井上　彰　（支持療法・緩和治療）

東北大学大学院医学系研究科医学統計学分野　教授
山口　拓洋（支持療法・緩和治療）

国立研究開発法人国立がん研究センター中央病院
支持療法開発部門　部門長
内富　庸介（全体監修）

研究協力者

国立研究開発法人国立がん研究センター
社会と健康研究センター健康支援研究部　部長
松岡　豊

聖マリアンナ医科大学　臨床腫瘍学講座　教授
中島　貴子

杏林大学医学部内科学腫瘍科　教授
長島　文夫

国立研究開発法人国立国際医療研究センター病院　薬剤部　薬剤部長
寺門　浩之

国立研究開発法人国立がん研究センター中央病院　薬剤部　薬剤部長

山口　正和

国立研究開発法人国立がん研究センター中央病院　薬剤部　副薬剤部長
橋本　浩伸

国立研究開発法人国立がん研究センター東病院
臨床研究支援部門研究企画推進部安全管理室　室員
米村　雅人

国立研究開発法人国立がん研究センター東病院　薬剤部　薬剤部長
川崎　敏克

静岡県立静岡がんセンター婦人科　医長
安部　正和

静岡県立静岡がんセンター消化器内科　医長
横田　知哉

国立研究開発法人国立がん研究センター東病院　緩和医療科　医長
松本　禎久

東京女子医科大学　看護学部　老年看護学　助教
川原　美紀

国立研究開発法人国立がん研究センター
社会と健康研究センター健康支援研究部　特任研究員
華井　明子

国立研究開発法人国立がん研究センター東病院
放射線治療科　特任研究補助員
嶋田　早苗

東京薬科大学　医療実務薬学教室　助教
川口　崇

国立研究開発法人国立がん研究センター
社会と健康研究センター健康支援研究部　特任研究員
東京大学大学院医学系研究科 臨床試験データ管理学講座　特任助教
宮路　天平

国立研究開発法人国立がん研究センター
社会と健康研究センター予防研究部　室長
島津　太一

国立研究開発法人国立がん研究センター
社会と健康研究センター健康支援研究部　室長
藤森　麻衣子

協力学会および評価担当者

日本癌治療学会　理事長　北川雄光
京都大学医学部附属病院　乳腺外科　戸井　雅和

日本がんサポーティブケア学会　理事長　田村和夫
弘前大学大学院　医学研究科腫瘍内科学講座　佐藤　温

日本緩和医療学会　理事長　細川豊史
帝京大学医学部　緩和医療学講座　有賀　悦子

日本サイコオンコロジー学会　代表理事　明智龍男
名古屋市立大学病院　緩和ケア部　奥山　徹

日本がん看護学会　理事長　雄西智恵美
群馬大学大学院　保健学研究科看護学講座　神田　清子

日本臨床腫瘍学会　理事長　南 博信
筑波大学　医学医療系臨床腫瘍学　関根　郁夫

日本放射線腫瘍学会　理事長　茂松　直之
新潟大学医歯学総合病院　放射線治療科　青山　英史

日本臨床腫瘍薬学会　理事長　加藤　裕芳
国立がん研究センター中央病院　牧野　好倫

日本癌学会　理事長　中釜　斉
国立うがん研究センター中央病院　上園　保仁

（順不同・敬称略）

目次

1. 支持療法・緩和治療領域研究ポリシーの目的
2. 支持療法・緩和治療の定義
  2.1 背景
  2.2 がん治療 Cancer treatment
  2.3 支持療法（supportive care for toxicities arising from cancer treatment）
  2.4 緩和治療（palliative care for symptoms arising from cancer）
3. 支持療法と緩和治療の研究特性
  3.1 支持療法の研究特性
    3.1.1 がん治療 cancer treatment と支持療法の関係
    3.1.2 がん治療 cancer treatment を行う治療科との協働関係
    3.1.3 介入の質
      3.1.3.1 薬物療法による介入
      3.1.3.2 薬物療法以外の介入
    3.1.4 有害事象と副作用
  3.2 緩和治療の研究の特性
    3.2.1 標準治療が未確立である場合が多いこと
    3.2.2 プロトコール治療の柔軟性
    3.2.3 対象とする患者の全身状態
    3.2.4 患者が治療を受ける場所
    3.2.5 インフォームド・コンセント
4. 研究対象集団
  4.1 試験対象と実際の臨床現場の患者の系統的な違い
  4.2 支持療法の研究対象集団
    4.2.1 対象集団および受けるがん治療 cancer treatment の多様性の許容
  4.3 緩和治療の研究における対象集団
    4.3.1 緩和治療の対象集団の定義
    4.3.2 対象集団の均一性と実施可能性のバランス
    4.3.3 年齢の規定
    4.3.4 全身状態の規定
    4.3.5 医師の予測による生命予後（Clinical prediction of survival: CPS）
    4.3.6 認知機能
    4.3.7 進行がん患者の定義
    4.3.8 臨床検査データ
5. 研究デザイン

5.1　検証的試験と探索的研究
5.2　支持療法の研究における研究デザイン
5.2.1　研究目的
5.2.2　検証的試験の研究デザイン
5.2.2.1　二重盲検ランダム化試験
5.2.2.1.2　既存治療対照二重盲検ランダム化試験
5.2.2.2　オープンラベルランダム化試験
5.2.2.2.1　エンドポイントについて盲検化したオープンラベルランダム化試験
5.2.2.2.2　生存などの客観指標を置いたオープンラベルランダム化試験
5.2.2.3　単アーム検証的試験
5.2.3　探索的研究の研究デザイン
5.2.3.1　検証的試験の前段階として行う探索的研究
5.2.4　説明的試験 explanatory trial と実践的試験 pragmatic trial
5.2.5　普及・実装研究
5.2.6　観察研究
5.3　緩和治療の研究における研究デザイン
5.3.1　研究目的
5.3.2　緩和治療の研究における研究デザイン
5.3.2.1　検証的試験
5.3.2.1.1　二重盲検ランダム化試験
5.3.2.1.1.1　プラセボ対照二重盲検ランダム化試験
5.3.2.1.1.2　既存治療対照二重盲検ランダム化試験
5.3.2.1.2　単アーム検証的試験
5.3.2.1.3　クラスターランダム化試験
5.3.2.1.4　実際の臨床現場での効果の確認（実践的試験）
5.3.2.2　探索的研究
5.3.2.2.1　検証的試験の前段階として行う探索的試験
5.3.2.2.2　探索的ランダム化試験
5.3.2.2.3　単アーム試験・観察研究
6.　エンドポイントと評価尺度
6.1　支持療法の研究におけるエンドポイントと評価尺度
6.1.1　プライマリーエンドポイント
6.1.1.1　がん治療 cancer treatment の治療成績に影響する急性毒性に対する支持療法
6.1.1.2　がん治療 cancer treatment による生活の質に影響する急性有害事象もしくは晩期有害事象に対する支持療法

6.1.2　その他のエンドポイント
6.1.2.1　がん治療 cancer treatment の成果を妨げないことを担保する
6.1.3　評価尺度
6.2　緩和治療の研究におけるエンドポイントと評価尺度
6.2.1　エンドポイントの選択
6.2.2　主要評価項目と副次評価項目の違い
6.2.3　苦痛緩和における臨床的に意味のある差（Minimum important difference：MID）
6.2.4　評価尺度
7.　対象患者の死亡に関する取り扱い
7.1　背景
7.2　安全性情報の管理
7.3　試験デザイン
8.　実施体制と品質マネジメント
8.1　基本的な考え方
8.2　研究の品質マネジメントとシステム（Quality Management System (QMS)）の構築 (30)
8.3　研究の品質管理（Quality Control; QC）
8.3.1　研究計画書（プロトコール）の作成
8.3.2　登録と割り付け（ランダム化）
8.3.3　データの測定、評価
8.3.4　研究の進捗管理、モニタリング
8.3.5　データマネジメント
8.3.6　臨床研究現場でのデータ収集プロセスの管理
8.3.7　統計解析
8.4　研究の品質保証（Quality Assurance; QA）
（参考文献）

1. 支持療法・緩和治療領域研究ポリシーの目的

このポリシーの目的は支持療法・緩和治療の臨床研究、特に臨床試験を行う際の指針を示すことである。すなわち、臨床研究における、①支持療法/緩和治療の定義、②研究の特性、③対象集団、④デザイン、⑤エンドポイントと評価尺度、⑥対象患者の死亡に関する扱い、⑦実施体制と品質マネジメントについてのポリシーを明らかにすることを目的としている。

なお、各専門領域における支持療法・緩和治療領域研究のあり方に差異があることを踏まえて、本ポリシーでは共通で用いるべき基本的な考えを示す。ポリシーは薬物療法や医学介入が前提であるが、一部の心理社会的介入、看護介入やリハビリテーションなど侵襲性の低い/ない臨床試験が本ポリシーに準じて計画されることも念頭に置いた。今回は支持療法・緩和治療の臨床研究ポリシーの基本部分の明確化を目的としたので、臨床研究法で扱う、「医薬品、医療機器等の品質、有効性及び安全性の確保に関する法律（薬機法）」による未承認薬・適応外の医薬品・機器などの臨床研究、製薬企業などから資金提供を受けて実施される臨床研究、臨床試験（治験）は扱わなかった。

2. 支持療法・緩和治療の定義

支持療法・緩和治療を定義する歴史と背景を述べる。

2.1 背景

近年、がん医療において疾患の治療のみならず、疾患を抱えた患者の苦痛の軽減や心理社会的支援も重視されるようになってきた。疾患に対する治療、すなわちがん治療 cancer treatment 以外の支援をまとめて表現するために、supportive care（サポーティブケア、支持療法）、palliative care（緩和ケア、緩和医療、緩和治療）といった表現が使用されているが、国際的にも厳密な定義はなく、日本語訳についても統一見解がない。したがって、ここでは質の高いエビデンスが求められている支持療法と緩和治療の臨床研究、特に試験を遂行するという観点から、必要最小限に定義することを目的としている。

Supportive care とは、もともとはがん治療領域においてがんに対する治療 cancer treatment に伴う合併症の予防・軽減（制吐や感染症対策など）を目的とした治療を指していた。しかし最近になって supportive care の示す意味がより広義に患者の quality of life を向上させる治療と支援を包括的に指すようになっており、その場合は、緩和ケアとの区別は実際難しくなっている。例えば、米国 National Cancer Institute の用語集(2017 年 11 月時点)では緩和ケアと同義に扱われ、がん治療による副作用の対策、がんによる症状の緩和、精神的ケア、病状や治療に関する情報の提供、併存疾患への対処、家族・介護者への支援、終末期の問題のすべてを包括する意味があるとされる。[1][2] 現在の supportive care という拡がりを持った広義の意味を考慮すると邦訳の支持療法の「療法」が意味する therapy や treatment では狭義となってしまい不適切となっている。

Palliative care の起源はホスピスにさかのぼる。ホスピスとはもともと 11 世紀ヨーロッパを中心に設けられた巡礼者や傷病者のための施設であり、ラテン語の hospitium（人をもてなすこと）を語源とする。その後、1967 年にセントクリストファーズホスピスが設立され、現代ホスピス運動が世界に発展した。1970 年代カナダのモントリオールに導入する際に、ホスピスという呼称はフランス語圏において貧しい人たちのための救済施設というイメージが強いため、palliative care と表現されたことが最初になる。さらに 1990 年に WHO が Cancer Pain Relief and Palliative Care を表明し、国際的な表現として定着した。現在は、「生命を脅かす疾患による問題に直面している患者とその家族に対して、痛みやその他の身体的問題、 心理社会的問題、スピリチュアルな問題を早期に発見し、的確なアセスメントと対処（治療・処置）を行うことによって、 苦しみを予防し、和らげることで、クオリティ・オブ・ライフを改善するアプローチ」（2002 年）と定義されている。(3)

Supportive care と palliative care は、広義に解釈すれば示すものが重複しており区別できない。今回、臨床研究、特に臨床試験を推進するという観点から、それぞれの中核部分を明確にするために意図的に狭い定義を用いるほうが、用語の示す内容が明確になるので望ましいと考えた。したがって、本ポリシーでは、Supportive care の当初の意味（がんに対する治療の合併症の予防と軽減を目的とした治療）を示すことが分かりやすいと考えられ、「支持療法**（supportive care for toxicities arising from cancer treatment)**」を用いることとした。一方、palliative care のうち薬物療法や侵襲的治療を対象とする場合は緩和医学（palliative medicine）と呼ばれることがある。今回のポリシーで念頭に置いている研究が薬物療法を含む医学的介入であることを明確にするために、敢えて「緩和治療**(palliative care for symptoms arising from cancer)**」と呼ぶこととした。これらは一般的な臨床概念として使用されている他の用語の存在を否定するものではない。

2.2　がん治療 Cancer treatment

がん治療 cancer treatment は、抗腫瘍効果 Disease-modifying effects を目的とした治療、すなわち、外科治療、がん薬物療法、放射線治療を指す。がん治療は cancer treatment の邦訳であるが、単に「がん治療」と記載すると薬物療法の「抗がん剤治療」と誤読されてしまう可能性が高いので、敢えて「がん治療 cancer treatment」と併記して記載していく*。

---

*脚注　「がん治療 cancer treatment」と会話で使用する際にも、薬物療法の「抗がん剤治療」と誤解されてしまう可能性が少なからずある。実際、支持療法の臨床試験の現場で混同され試験計画書上「抗がん剤治療」と「がん治療 cancer treatment」が取り違えられ重大な逸脱、違反や事故につながる可能性がある。そこで、誤解のリスクを避ける対策として、支持療法の臨床試験を行う現場ではがん治療 cancer treatment を敢えてがんに対する「本体治療」と呼んで会話し、試験計画書では「がん治療（本体治療)」と記載することを勧めている。

2.3　支持療法（supportive care for toxicities arising from cancer treatment）

がん治療 cancer treatment で発生する副作用に対して予防もしくは症状軽減を目的として行う治療を指す。副作用には、治療後の合併症や後遺症を含む。

例えば、化学療法誘発性悪心・嘔吐（chemotherapy-induced nausea and vomiting: CINV）、発熱性好中球減少症（febrile neutropenia: FN）、化学療法誘発性末梢神経障害（chemotherapy-induced peripheral neuropathy: CIPN）、放射線による皮膚炎、術後創部痛、術後せん妄などに対する治療を指す。

2.4　緩和治療（palliative care for symptoms arising from cancer）

腫瘍の影響によって生じた苦痛や症状に対して予防もしくは症状緩和を目的として行う治療を指す。

例えば、がん疼痛（cancer pain）、がん性腹膜炎による消化管閉塞（malignant bowel obstruction: MBO）、がん関連倦怠感（cancer-related fatigue: CRF）、がんによる呼吸困難に対する治療などを指す。

3.　支持療法と緩和治療の研究特性

支持療法と緩和治療の臨床試験は、一般の臨床試験と比較して、共通する部分は多いが、相違点や特徴について重要点を特記する。

3.1　支持療法の研究特性

支持療法の臨床試験は原則としてがん治療 cancer treatment を含む一般の臨床試験と共通する方法論に沿って行うが、以下のような特性がある。

3.1.1　がん治療 cancer treatment と支持療法の関係

支持療法はがん治療 cancer treatment で発生する副作用に対する治療なので、対象とするがん治療 cancer treatment が将来も広く臨床で使用されるとの予測のもと新規の支持療法の研究計画を立てることになる。もし、現在のがん治療 cancer treatment 自体が使用されなくなった場合、付随する支持療法もなくなることを意味するので、計画する際は慎重に計画するようにする。

3.1.2　がん治療 cancer treatment を行う治療科との協働関係

支持療法の臨床試験を行う場合、がん治療 cancer treatment を行う科(以下、診療科)と支持療法を行う科がしばしが異なる。その場合、がん治療 cancer treatment を行う診療科との強固な協働関係なくして支持療法の研究は成立しない。例えば、「外科手術後の誤嚥性肺炎を低減するための術前専門的口腔ケア介入」という試験の場合にはがん治療 cancer treatment を行う治療科は外科であり、支持療法を行う科は歯科になる。

支持療法の臨床試験を計画する立案段階から治療科の研究者を研究グループに迎え、がん治療 cancer treatment の治療科が中心的役割の一角を担えるよう共同研究者として協働

関係を構築する必要がある。臨床試験の開発スピード、患者のリクルート、研究成果の普及・実装のすべての段階に大きく影響するからである。

3.1.3　介入の質

3.1.3.1　薬物療法による介入

薬物療法による介入の場合には通常の臨床試験と同様に支持療法に用いる薬物の投与時期、投与開始規準、延期、中止規準などをあらかじめ定め、術者裁量の範囲がいたずらに広くならないように留意することで最低限の質を担保する。

3.1.3.2　薬物療法以外の介入

支持療法の介入は、薬物療法だけではない。例えば、看護ケアや運動療法などの非薬物介入では、患者と医療者の双方が関わって成立する場合がある。非薬物介入であってもその質を一定水準以上に保たなくては試験結果の信頼性が損なわれる。

一定の水準に達している術者のみが参加できる制度を作ったり、試験の開始前に試験参加施設の医療者に実技指導を行ったりすることで、介入の質の担保を行う。患者・家族の協力が必要な場合にはプロトコールや説明同意文書以外にも詳細な実施手順書を作成し、介入の質を一定水準に保つ必要がある。

3.1.4　有害事象と副作用

有害事象とは、医薬品の使用、放射線治療、または手術等と時間的に関連のある、好ましくない、意図しないあらゆる徴候（例えば、臨床検査値の異常）、症状、または疾病のことであり、当該治療との因果関係の有無は問わない。副作用は、有害事象のうち医薬品だけでなく、近年では CTCAE ver3.0 以降手術の合併症や放射線治療などすべての治療との因果関係が否定できないものをいう。

支持療法の研究ではもともと頻度の高い、重篤な副作用が生じるがん治療 cancer treatment を対象に行われることが多い。出現する有害事象にはがん治療 cancer treatment の副作用に由来するものと、支持療法自体の副作用に由来するもの双方が想定されるので、由来を区別して記載できるようプロトコールに含める。

3.2　緩和治療の研究の特性

緩和治療を受ける患者は、がん治療 cancer treatment 終了前後の対象が多くなり脆弱であることが多い。臨床試験に組み入れられる対象と実際の臨床現場の患者集団が大きく異なる場合には有用な知見が得られる研究デザインを念頭に置くことが必要である（図 1b 参照）。この他、緩和治療の臨床研究実施上重要な点を特記する。

3.2.1　標準治療が未確立である場合が多いこと

現状、緩和治療ではある苦痛に対応して行うことが勧められる治療が 1 つに限定されていない。これは、がん治療 cancer treatment と異なり、ある苦痛に対する標準治療として緩和治療が確立していないためである。現状としては標準治療を確立する手掛かりとなるような研究が主であり、標準治療とみなされる治療がない場合が多いことを認識する必要がある。

3.2.2　プロトコール治療の柔軟性

緩和治療の特徴として、現在行っている治療の効果が短期間（数時間から数日間）に明確になるため、患者の状況に応じた治療の頻回の変更が実際の臨床現場では可能であることが多い。がん治療 cancer treatment の効果は開始後直ぐには判断できず最終的には数か月から数年という生存期間として評価されるのに対して、緩和治療の効果は数時間から数日間で評価でき、患者の状態に合わせて治療を頻回に修正することが可能であることが多い。

この点は、実際の臨床現場の治療に合わせて、プロトコール治療の減量・延期・中止規準の設定を柔軟にできることを含める必要があることを意味する。例えば、鎮痛効果が得られる投与量まで患者の状態に応じて投与量を調節（titration）する、という場合である。[(4)(5)]

また、研究課題の設定という探索段階では、介入が完全に標準化されていなくても、日々の治療の変更の指針となる情報を得る研究にも意義があることを意味する。例えば、粘膜吸収性フェンタニルとモルヒネ注射薬の比較など異なる鎮痛薬の相対的な効果を探索する、という場合である。[(6)]

3.2.3　対象とする患者の全身状態

緩和治療の試験対象は、患者の予後が短い場合が多い。そのため、①研究を完遂できる対象を設定すること、②研究の実施可能性を予備試験で評価すること、③研究期間は可能な限り短く設定すること、および、④患者の予期された死亡の扱いをあらかじめ決めておくことが重要である。

3.2.4　患者が治療を受ける場所

緩和治療の臨床研究の対象者は病院の特定の診療科に限らず、病院全体・地域全体に存在する。したがって、実際に試験を実施する場所において該当する患者候補がどれだけいて、どれだけ登録されるのかの見積もりを得ることは極めて重要である。

3.2.5　インフォームド・コンセント

病状進行とともに認知機能障害を合併する頻度が増加する。そのためしばしば、患者個人から直接インフォームド・コンセントを得ることが不可能な場合がある。事前の同意（in-advance consent）や代諾者による同意を慎重に検討する必要がある。

4.　研究対象集団

支持療法・緩和治療の研究においては研究結果が実際の臨床により良く普及・実装されるために以下のことを考慮することが勧められる。

4.1　試験対象と実際の臨床現場の患者の系統的な違い

支持療法においても緩和治療においても、臨床試験が行われる患者集団と実際に治療を受ける患者集団との間に系統的な差が生じてしまう場合がある。

臨床試験の対象を狭くした場合、実際の臨床現場と比べて全身状態の良い患者のみを対象として試験を行うことになり、試験の結果があてはめられる実際の患者は少なくなる（図 1a）。すなわち、外的妥当性が低いことになる。逆に、実際の臨床現場への出口を過剰に意識しすぎて臨床試験の対象を広くした場合、支持療法では患者背景がばらついて効果が希釈され試験結果の解釈を困難とするリスクを負ってしまうし、緩和治療では全身状態の不良な患者が試験を受けることになり試験の遂行そのものが困難となるリスクを負うことになる（図 1c）。すなわち、内的妥当性が低いことになる。

したがって、支持療法・緩和治療の臨床試験においては、理想的な患者集団（ideal world）と実際の臨床の患者集団（real world）の差異を意識しつつ適正な対象集団を設定する必要がある（図 1b）。臨床試験を実施して検証するに留まらず、検証後には当該治療が実際の臨床現場で実施される患者集団（臨床試験からはしばしば除外される患者集団）を対象とした観察研究や Big data を用いた調査を行うことにより、臨床試験の結果が実際の臨床の患者に反映されているかを監視する必要がある。

図 1　支持療法と緩和治療の臨床試験の対象

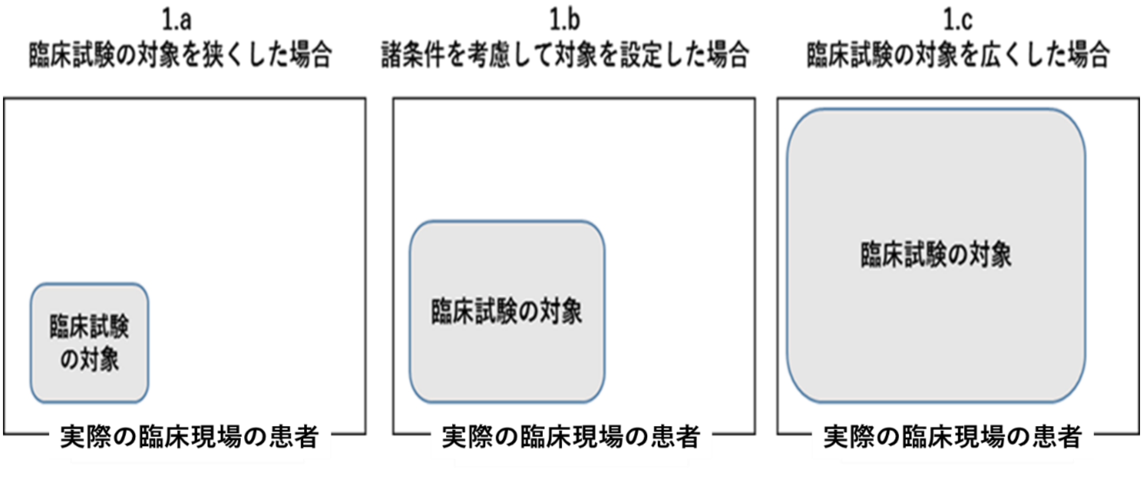

4.2　支持療法の研究対象集団

4.2.1　対象集団および受けるがん治療 cancer treatment の多様性の許容

試験の目的が効能 efficacy の場合、できる限り均一な背景を持つ対象集団を選定して、効能が希釈されないよう努力する（図 1a）。一方で、試験結果が吟味された後、可及的速やかに実際の臨床現場で広く実践されることを考慮すると、その場合の対象集団はがん治療 cancer treatment 自体の内容（薬剤、手技）が多少置き換えられたとしても試験結果が外挿できるようにある程度の幅をもった基準を設定することが望ましい（図 1b）。

例えば、ある薬剤(薬剤A)に固有の毒性を管理するための新規支持療法の研究を計画する場合、特定の疾患/薬剤双方の適格基準を厳しく規定するのではなく、薬剤Aを使用する複数のがんの部位を適格基準に組み入れる、さらに/もしくは、薬剤Aを使用する複数のレジメンを許容する。例えば、ある制吐剤が中等度以上の催吐作用を有する化学療法の支持療法として有用であることを検証する際には、単一の疾患やレジメンに限定するのではなく、該当する複数のレジメンを用いる複数のがんの部位を対象とした試験デザインを組むのが好事例である。

手術に対する支持療法の研究では、特定の疾患と特定の術式双方を厳しく規定適格基準とするのではなく、同じ程度の侵襲の手術を適格にする、もしくは、手術操作の加わる場所が同一であれば術式は問わないなど適格基準の幅を許容することを考慮する。試験運営上適格基準の拡大が難しい場合は、試験の出口戦略としての疾患や手技/レジメンなどの適応拡大を議論しプロトコールにそのプロセスを記載する。

最終的に、「得られた知見が広く実際の臨床現場でより良く普及・実装される」ことが支持療法における臨床試験の重要点であることを念頭に置くべきである。

4.3　緩和治療の研究における対象集団

4.3.1　緩和治療の対象集団の定義

緩和治療の研究においては、そもそも臨床試験の対象となる集団の診断基準が明確になっていないことがしばしばある。しかし、可能な限り、国際的なコンセンサスのある診断基準に基づいて対象を定義する。例えば、神経障害性疼痛の診断基準は International Association for the Study of Pain (IASP) の定義[7]、うつ病の診断基準は Diagnostic and Statistical Manual of Mental Disorders（DSM）[8]に基づいて行うことができる。

一方、対象集団の診断基準に関する国際的なコンセンサスがない場合、先行研究を参考にし、当該領域の複数の専門家の合意をもとに、客観性と再現性があるように対象集団の定義を記載する。介入研究を計画する前には、観察研究として該当する診断基準の信頼性や妥当性を確認することが望ましい。対象集団の定義は、先行研究との比較可能性や研究の結論に大きく影響するため、研究者独自の定義を用いてはならない。

4.3.2　対象集団の均一性と実施可能性のバランス

どの臨床研究領域においても、対象集団をなるべく均一にすることが研究知見の結果を個々の患者に還元するうえで重要である。対象集団が不均一であると、効果が希釈・相殺されやすい。例えば、がん疼痛の患者全体を対象とするよりも、膵臓がんによる内臓痛、特定部位の神経障害性疼痛の患者のみを対象とするほうが患者集団としては均一になる。

一方、均一な集団のみを対象とした臨床試験を計画すると症例集積が実際上不可能になることも多い。したがって、研究立案の上では可能な限り患者集団を均一にするべきであるが、実施可能性を維持することとのバランスをとることが重要である。

対象集団が不均一である場合には、予備的な知見に終わってしまう場合であってもサブグループでの解析が可能なように特徴となる背景要因を取得しておく。

4.3.3　年齢の規定

緩和治療を実施する実際の集団で高齢者が多いこと、暦年齢だけで患者の脆弱性は判断できないこと、実際に臨床試験を実施する際に研究対象とすることがふさわしくない患者は医師の判断で対象とされないことから、緩和治療の研究においては、年齢の上限は患者集団を規定する必須の条件としない。ただし、認知機能を主要評価項目とする研究など、アウトカムや介入に年齢の関与が大きいことが予測される場合には、年齢の規定を設けることを検討する。

また年齢の下限に関して、未成年者のインフォームド・コンセント取得過程においては特別な配慮が必要であるが、他の臨床研究と同様である。

4.3.4　全身状態の規定

年齢と同じように全身状態についても、全身状態の不良な患者が緩和治療の主な対象である。したがって、緩和治療を受ける患者を反映する集団を対象として選択するという観点からは、全身状態の悪化した患者も対象にするべきである。一方、臨床試験においては試験治療が終了するまでに死亡する可能性が高い患者をあらかじめ除外しておく必要もある。このため、全身状態の評価が必要と考えられる場合、医師の予測による生命予後（Clinical prediction of survival: CPS）(9)を基準とし、「余命が 1 ヶ月以上あると見込まれる患者」などと表現する。

CPS は主観的な基準であるため、より客観的な基準として解釈しやすい performance status（ECOG PS(10), KPS(11)）をできるかぎり条件に含める。

また、どのような患者に臨床試験が行われたのかを明示するために、患者の全身状態に関する情報として予後予測指標を取得することが望ましい。

4.3.5　医師の予測による生命予後（Clinical prediction of survival: CPS）

CPS とは、担当医の臨床的な判断から経験的に予測された患者の生命予後を指す。医師の予後予測は系統的に楽観的であることが分かっている。すなわち、CPS を用いたとしても試験を受けた患者の相当数では、実際の生命予後は予測された生命予後より短い。したがって、将来的には、臨床試験の患者集団を表現するための客観性のある指標が必要である。客観的な生命予後指標として、Palliative Prognostic Score（PaP score）(12)、Palliative Prognostic Index（PPI）(13)、Prognosis in Palliative care Study predictor models（PiPS models）(14)、Palliative care Phase(15)などがあるが、これらは臨床試験で用いられた経験が少ない。当面、全身状態の規定としては CPS を基準として用いることとし、できる限り performance status（ECOG）を併記する。

4.3.6　認知機能

患者の主観的評価をエンドポイントとする緩和治療研究において、対象の認知機能を評価することは重要であり、原則、Mini-Mental State Examination（MMSE）(16)等を使用することが勧められる。ただし終末期の呼吸困難に関する緩和治療の研究など、切迫した状況での認知機能検査を用いた評価を行うこと自体が不適切であることが自明の場合もある。し

たがって、患者の主観的評価をエンドポイントとする緩和治療研究において、「インフォームド・コンセントが可能である」と研究者の観察から患者の一定の判断能力があるとみなしてよい場合がある。

4.3.7　進行がん患者の定義

緩和治療の患者集団を定義するときにしばしば進行がん患者を定義する必要がある。かつては terminally ill cancer patients や incurable cancer patients と表現されることがあった。しかしこれらの表現は定義があいまいでありすすめられない。ほとんどの場合、「遠隔転移があるまたは局所進行がん患者（metastatic or locally advanced cancer patients）」と定義することによって、対象集団が記述できる。がん種が特定されている場合は、「臨床病期IV期の肺がん」のように限定してもよい。

4.3.8　臨床検査データ

緩和治療の実際の臨床現場では、臨床検査データが治療を行う基準とならないことも多いが、試験薬の代謝/排泄に関連するなど治療を行ううえで臨床試験の安全性を最低限担保するための項目（腎排泄の試験治療薬の場合の腎機能など）は取り込む必要がある。対象となる患者の状態を想定し、データ採取の侵襲性を鑑みて採取項目等を設定することが望ましい。

5.　研究デザイン

支持療法・緩和治療の臨床試験における研究デザインの概要を示す。研究方法の詳細な手順については別途各論を作成する。研究デザインについて支持療法・緩和治療に共通した部分もあるが、ここでは支持療法で特に留意する点は支持療法の項に、緩和治療で特に留意する点は緩和治療の項に分けて記載した。

5.1　検証的試験と探索的研究

支持療法・緩和治療の研究では、通常診療ですでに使用されている薬物・用法用量が評価の対象となる場合が多く、新薬を除いては治療開発で使用される Phase I/II/III という呼称がなじまないことが少なくない。したがって、検証的試験、探索的研究という観点から試験を分類し整理する。

検証的試験とは治療の有用性を確立することを目的として行われるものである。これ以外の研究は探索的研究として区別する。(図 2)

図 2.　検証的試験と探索的研究

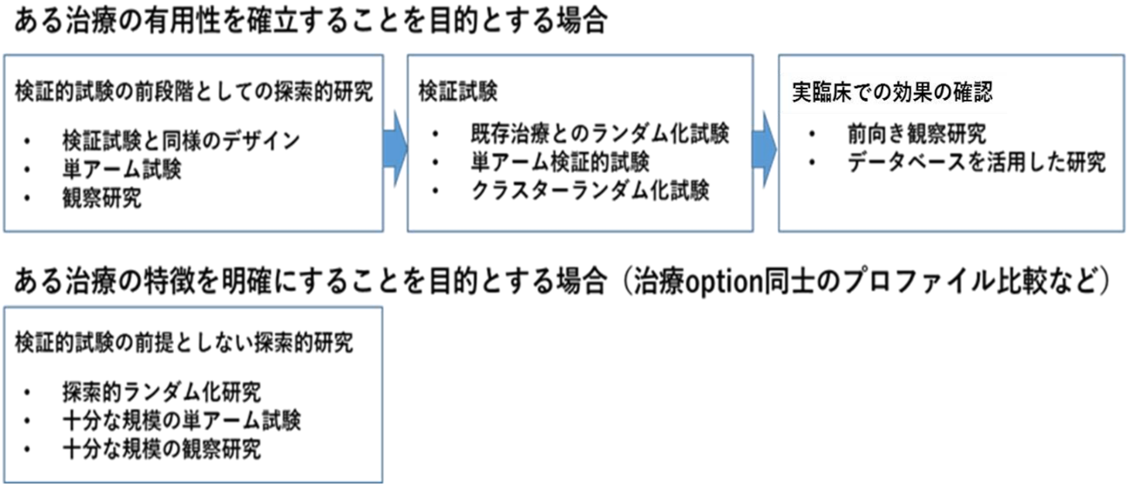

5.2　支持療法の研究における研究デザイン

5.2.1　研究目的

支持療法はがん治療 cancer treatment で発生する副作用に対して予防もしくは症状軽減を目的として行う治療である。その結果、がん治療 cancer treatment のポテンシャルを最大限に引き出し治療成績の向上に資することが期待できる（生存期間の延長）。同時に、がん治療 cancer treatment 中や終了後の日常生活を阻害する要因を予防、または軽減することも期待できる成果の一つである（QOL の維持・向上）。

支持療法研究ではそれらを反映する指標をエンドポイントに置いて上記の目的を達成しているか評価する必要がある。

5.2.2　検証的試験の研究デザイン

支持療法研究で新規の介入が実際の臨床現場で実施されるに足るか否かを評価するためには、無治療もしくは現在の標準治療を対照群としたランダム化検証的試験を行うことを原則とする。支持療法では結果の解釈において客観性が重視されるため、プラセボ、中央判定、判定者の盲検化など客観性の保持に努める必要がある。

5.2.2.1　二重盲検ランダム化試験

対照群にプラセボを用いて盲検化を行う。支持療法研究は他の臨床研究と方法論は同じであり、このデザインによる標準治療の決定が理想的である。

一般の腫瘍学での臨床試験と比べて支持療法の検証的試験ではエンドポイントの客観性が乏しい場合がある。そういった場合には評価の中立性を担保するためにも二重盲検ランダム化が強く奨められる

5.2.2.1.2　既存治療対照二重盲検ランダム化試験

既存治療として、標準治療が確立していると考えられる場合には、標準治療を対照とした

比較試験を実施する。

標準治療としてのコンセンサスは得られていなくてもすでに一般的な治療法があり、新しい治療の効果がそれを上回るかに関心がある場合には、既存治療を対照とした比較試験を実施する。この場合、原則、既存治療はプラセボと比較して効果があることが確認されている必要がある。

5.2.2.2　オープンラベルランダム化試験

5.2.2.2.1　エンドポイントについて盲検化したオープンラベルランダム化試験

対照群と試験群が明らかに異なる場合（理学療法や皮膚炎塗布剤など）は、オープンラベルで試験を行う。オープンラベルでの臨床試験は症例を登録した研究者のエンドポイント評価にはバイアスがかかるため、盲検化された評価者が中央判定を行うことが必須である。支持療法領域ではその領域に標準的介入がなく、その結果、対照群が無介入ということもありうるが、そういった場合でも対照群を無介入としたランダム化試験として行うことが望ましい。無介入が許容できない場合には通常の介入群 treatment as usual(TAU)と試験介入群といった実際の臨床現場に即したデザインも許容される。ただし、差を検出しづらくなることも留意すべきである。

5.2.2.2.2　生存などの客観指標を置いたオープンラベルランダム化試験

全生存割合や中央判定を用いた無増悪生存割合/無再発生存割合などバイアスのかかりづらい指標を置く場合にはオープンラベルランダム化試験をもって検証的試験とみなす。

プライマリーエンドポイントが客観指標であっても重要なセカンダリーエンドポイントに主観的な要素が強い場合にはセカンダリーエンドポイント以下も盲検化された評価者が中央判定を行うことが望ましい。

5.2.2.3　単アーム検証的試験

単アームの試験を原則、検証的試験として行わないが、無治療との比較が倫理的に困難なやむを得ない事情を有するうえ、他に候補となる介入方法が存在しないといった特殊な状況に限定して十分な症例数を用いた単アーム試験を検証的試験に位置付けることがある。

例えば、同じ手技を新しい機器で行うことにより今まで以上の有用性が示せるか、または従来のものより悪くならないかを確認する試験を想定する。従来の機器を改善した新しい機器が実際の臨床現場で既に販売、浸透している場合、今更従来の機器を持ち出してランダム化試験を実施することは現実的ではない場合、単アームで評価項目を設定して確認することはありうる。

5.2.3　探索的研究の研究デザイン

5.2.3.1　検証的試験の前段階として行う探索的研究

検証的試験の前段階として行う探索的研究は、検証的試験を行うために必要なデータを収集することを目的とする。安全性や有効性について評価を行うことは基本的には従来の

試験と同じ方法論に従うが、それに加え、試験自体の実施可能性（対象患者の集積能力、プロトコールの遵守率など）についても評価する必要がある。

支持療法では他科との連携などは実際に試験を行わないとわからない施設のバリアも多くあり、検証的試験で多施設共同試験を想定している場合にはまず単施設もしくは少数施設で他科連携を行い試験の実施可能性を見るということも探索的試験の目的となる。詳細な手順書、研究実施者への訓練、実施後の遵守評価はこの段階で詳細に作成する。

複数のがん治療 cancer treatment に関する大規模な臨床試験での付随的な情報として十分な有害事象のデータがあり仮説を裏付けられるのであれば、探索的な前向き試験を省略できる場合がある。

5.2.4　説明的試験 explanatory trial と実践的試験 pragmatic trial

前述の検証的、探索的試験に対して、説明的、実践的試験という分類機軸も存在する。説明的試験は、理想的な環境における効能（efficacy）を評価するのに対し、実践的試験は、実際の臨床現場における効果（effectiveness）を評価することを目的とする。説明的試験と実践的試験は、はっきり分けられるものではなく、連続したものである。実践的試験の特徴は、適格基準、参加者募集にかける努力、介入の場（プライマリケアか、専門診療かなど)、組織の資源・医療従事者の専門性、医療従事者への介入導入方法、介入プロトコール遵守度、追跡や測定の強度が実際の臨床現場に近いものである。また、プライマリアウトカムは患者にとって重要で、実際の臨床現場で用いられるものである。主解析ではすべてのデータを用いる（intention-to-treat analysis)。[17]

支持療法に関する臨床試験では多職種連携が成熟した施設にて行われるため、施設間格差による一般化可能性が大きな問題となる。そのような場合は、実践的試験による効果検証を行うことを推奨する。検証的研究を単アームで行った場合や条件の整った数施設のみで実施された場合には、さらに実践的試験を行うことを推奨する。

5.2.5　普及・実装研究

有効性に基づき推奨されているが、実際の臨床現場で広く普及していない介入法を実装するための研究。介入法が普及しない背景を明らかにし、患者、家族、医療従事者、施設の意思決定者、環境、政策などにアプローチする複数の実装戦略を用い、有効な介入法を日常診療として広く採用され、その状態が維持されることを目的とする。[18]

5.2.6　観察研究

得られた知見が実際の臨床現場に適切に影響を与えているかについて対象施設をより広げた前向き観察研究や学会員を対象とする単位での支持療法の利用に関する実態調査、DPC データを用いた調査などの実施が奨められる。

5.3　緩和治療の研究における研究デザイン

5.3.1　研究目的

緩和治療の研究に共通する目的は、患者の苦痛の軽減に資する知見を提供することである。個々の研究としては、「ある治療の効果を確立すること（検証的試験）」と、「ある治療の特徴を明らかにすること（探索的研究）」とがある（図 2)。

原則的には、全ての緩和治療について、効果を確立するための検証的試験を行うことが理想である。しかし、3.2 に述べた緩和治療の特徴から考えて、検証的試験のみが臨床に資する研究ではなく、すべての検証的試験が実施可能というわけでもない。臨床疑問によっては、「ある治療の特徴を明らかにすること」によって臨床家の判断に資する研究とすることができる。

例えば、粘膜吸収性フェンタニルとモルヒネ注射薬の比較など異なる鎮痛薬の効果の相対的な効果を探索する試験などが相当する。[19]
すでに一般的に行われている治療の特徴がある程度明らかになれば臨床の実践上有用であると考えるならば、治療の特徴を明らかにすることを目的とした観察研究も価値がある。また、脆弱な患者集団を対象とした研究課題では、きわめて限定された患者集団を対象とした検証的試験よりも、治療対象となりうる全患者を対象とした観察研究を行うほうが臨床疑問に合致した知見が得られる場合がある。

例えば、死亡直前期の治療抵抗性の苦痛に対する鎮静や輸液などが該当する。[20][21][22]
臨床疑問に応じた研究目的を達成できる研究デザインを用いることが重要である。

5.3.2　緩和治療の研究における研究デザイン

5.3.2.1　検証的試験

5.3.2.1.1　二重盲検ランダム化試験

一般の腫瘍学での臨床試験と比べて緩和治療の検証的試験ではエンドポイントの客観性が乏しい。そのためエンドポイント評価の中立性を担保するためにも基本的には二重盲検ランダム化が必須と考えられる。

5.3.2.1.1.1　プラセボ対照二重盲検ランダム化試験

治療薬の効能 efficacy を検証する場合、検証的試験は二重盲検プラセボ対照ランダム化試験で行う。

自覚症状の変化には、時間経過、試験の対象とする緩和治療以外の効果、疾患や病態に対する治療の効果、心理社会的支援の効果、プラセボ効果など複数の要因が影響するためである。プラセボの使用に当たっては、患者の不利益にならないように、試験の対象としている治療の効果が確立していないこと、十分なインフォームド・コンセントを得ること、苦痛が悪化した場合の救済策（レスキュー薬の使用など）が取られていることが必須である。すべての患者が治療を受けることができるという点から fast-track study（介入を受ける時期によってランダム化を行う試験）の可能性を検討する。

治療薬が、緩和治療薬としての開発段階の場合や、他領域において薬効があることがすでに確立している場合には、特定集団での再現性をみる目的でない限りプラセボ対照試験は必要ない。

5.3.2.1.1.2　既存治療対照二重盲検ランダム化試験

既存治療として、標準治療が確立していると考えられる場合には、標準治療を対照とした比較試験を実施する。

標準治療としてのコンセンサスは得られていなくてもすでに一般的な治療法があり、新しい治療の効果がそれを上回るかに関心がある場合には、既存治療を対照とした比較試験を実施する。この場合、原則、既存治療はプラセボと比較して効果があることが確認されている必要がある。

5.3.2.1.2　単アーム検証的試験

すでに外国の知見から国内のガイドラインで推奨されているなどの理由でランダム化試験が実施できないことがある。その場合、よくデザインされた単アーム試験を検証的に行う。その際には均一な対象患者を設定し、適切な閾値・症例数設定などを行うことが必要である。

5.3.2.1.3　クラスターランダム化試験

クラスターランダム化とは患者を一単位としてランダム化するのではなく地域や施設をひとつの単位(クラスター)としてランダム化した研究である。集団単位として実施される治療法などの比較を行う場合には、クラスターランダム化試験がとりうる試験デザインの一つとなりうる。例えば、ある苦痛に対するクリニカルパスや治療アルゴリズムの効果全体を見る場合などが該当する(23)。

5.3.2.1.4　実際の臨床現場での効果の確認（実践的試験）

緩和治療では、検証的試験の対象となる患者と実際の臨床現場の患者の状態に差があることが多い（図１）。そのため、検証的試験を終了した後、十分な規模の前向き観察研究やデータベース研究を実施して効果と安全性を確認するよう、計画段階から熟慮することが望ましい。

5.3.2.2　探索的研究

記述研究・探索的研究には、検証的試験で取り組むべき仮説を明確にする（hypothesis gathering）ための研究と、記述的な結果を得ること自体が目的の研究とがある。

5.3.2.2.1　検証的試験の前段階として行う探索的試験

検証的試験の前段階として行う探索的試験は（図２）、検証的試験を行うために必要なデータを収集することを目的とする。当該の治療効果の推定に加えて、想定している検証的試験のデザインの実施可能性、エンドポイントの選択の妥当性の根拠を得ることなどを目的として行う。緩和治療は毒性についての探索（安全性の確認）が課題となることは少ないが、必要な場合には安全性の評価を目的とする。

この段階では、研究の実施可能性について評価することが重要である。実施可能であるとは、想定する患者がたしかに存在し登録される（登録)、研究施設において研究が無理なく実施できる（遂行)、患者が研究を完遂できる（完遂）ことを意味する。緩和治療の多くの臨床試験で患者が想定より登録されなかった、もしくは完遂できなかった事例があったことから考えて、検証的試験を実施する前に実施可能性を明らかにしておくことが重要である。

あわせて、検証的試験で使用するエンドポイントの選択（取得時期、取得方法なども含む）の妥当性を評価する。どのようなエンドポイントが意味のある変化を生じるか、取得時期はいつが適切か、誰がどうやって取得することが適切かを評価する。探索的研究では想定している検証的試験よりも多くのエンドポイントを複数の測定ポイントで取得することが一般的である。

試験デザインは、本来であれば、実施可能性の評価の点からも検証的試験と同様のデザイン（通常はランダム化試験）をとることがすすめられる。しかし、試験が実際上実施できない場合には、単アーム試験や観察研究の十分な検討などで代用できる場合もある。用法用量について海外でのエビデンスを外挿する際、本邦での実際の臨床現場と大きく乖離がある場合には単アームで実施可能性試験（feasibility study）を行う。

5.3.2.2.2　探索的ランダム化試験

緩和治療領域ではある一つの病態に対して複数のアプローチが存在し、一つに決める必要がない状況がある。そういった状況では、各治療で効果や副作用のプロファイルに臨床的意義のある差異が存在するかについて評価するためにランダム化試験を行う。例えばある病態に対する薬 A と薬 B があり、どちらも使用されているが薬 A は吐き気が多く出るが薬 B は不眠が多く出るかもしれないなどの仮説に対して臨床的に意義があるほどの差異であれば吐き気を嫌う患者は B を、不眠が一番の悩み、という患者は A を選択することになる。このように患者の意思決定にかかわる情報を補完することや今後の研究課題の明確化など目的に行われる[24]。

5.3.2.2.3　単アーム試験・観察研究

ランダム化試験が理論上実施できない場合（例えば、非常に強い苦痛を持った患者に対して当該治療しか対応がないと考えられる場合)、あるいは、ランダム化試験の計画は可能だが実施可能性が非常に低い場合（例えば、死亡が差し迫った患者に対するプラセボ試験）においては、検証的試験を前提とせずに、単アーム試験や観察研究から効果の推定を行うことが妥当である場合も考えられる。

6. エンドポイントと評価尺度

支持療法・緩和治療を計画するうえでのエンドポイントの考え方をまとめる。共通した留意点もあるが、ここでは支持療法で特に留意する点は支持療法の項に、緩和治療で特に留意する点は緩和治療の項に分けて記載した。

6.1 支持療法の研究におけるエンドポイントと評価尺度

支持療法の特性から以下の点について留意する。

6.1.1 プライマリーエンドポイント

6.1.1.1 がん治療 cancer treatment の治療成績に影響する急性毒性に対する支持療法

支持療法はがん治療 cancer treatment で発生する有害事象に対して予防もしくは症状緩和を目的とし、その結果、がん治療 cancer treatment のポテンシャルを最大限に引き出し治療成績の向上に資することが理想である。そういった観点から、全生存期間など生存に関する指標がプライマリーエンドポイントの候補にあがるが、実際に治療成績に影響する主な要因はがん治療 cancer treatment の内容に帰属することが多い。その場合、全生存割合や無再発生存割合で支持療法そのものの効果を評価することが難しい。

支持療法によりがん治療 cancer treatment の治療成績向上が想定される場合においても、生存期間ではなく、がん治療 cancer treatment のサロゲートエンドポイントであるがん治療 cancer treatment の完遂率をプライマリーエンドポイントに設定する。研究期間短縮という観点から現実的である。具体例を挙げる。

例）放射線治療毒性に対する支持療法：がん治療 cancer treatment の完遂割合をプライマリーエンドポイントに設定する。

放射線治療を 6 週間以内に完遂できた患者の予後が、完遂できなかった患者と比較して有意に良好であることが分かっている場合、完遂できない理由の半数が急性有害事象によるものであったと仮定する。

→新規の支持療法を導入することで急性有害事象の頻度を下げる仮説を立てる。

→急性有害事象の頻度が下がれば完遂割合が向上する。

→完遂割合が向上すれば治療成績の向上に寄与する可能性がある。

→エンドポイントを生存期間のサロゲートエンドポイントである治療完遂割合に設定する。

6.1.1.2 がん治療 cancer treatment による生活の質に影響する急性有害事象もしくは晩期有害事象に対する支持療法

治療終了後の日常生活に支障をきたす有害事象を予防または軽減する目的の支持療法では生存期間に関連するエンドポイント以外のものを設定することもある。ただし、比較可能かつ非専門家も納得する客観性、再現性を有するエンドポイントを設定しなくてはいけな

い。

6.1.2　その他のエンドポイント

6.1.2.1　がん治療 cancer treatment の成果を妨げないことを担保する

有害事象を抑えることを目的とした支持療法において、しばしばがん治療 cancer treatment の効果を減弱してしまった結果有害事象の発生が抑えられたという可能性を加味しなければならない。支持療法そのものががん治療 cancer treatment に対してもたらす負の影響が懸念される場合には、コプライマリーエンドポイントとして生存期間に関連する指標を設定する必要がある。

コプライマリーエンドポイントとは 2 つ以上同時に達成すべき項目である。例えば有害事象 Gr.3 発生割合と全生存割合（OS）をコプライマリーエンドポイントに設定した場合、有害事象 Gr.3 の発生割合が有意に抑えられ、かつ OS が一定水準以上を確保しているということを示されて初めて新規の支持療法として認められることになる。

例えば、コプライマリーエンドポイントを設定すべき臨床研究の例としては、放射線治療の有害事象を軽減するための放射線防護剤(アミフォスチン)、抗がん剤治療の骨髄抑制を緩和するための事前輸血/エリスロポエチン投与などがある。

ただし、上記は適格となるがん治療 cancer treatment や対照群が単一でない場合には設定が難しい。がん治療 cancer treatment の効果に対して負の影響が懸念される支持療法を研究課題にする場合には、対象を限定することも考慮すべきである。

6.1.3　評価尺度

有害事象の評価には NCI Common Toxicity Criteria of Adverse Events（CTCAE）[25]をはじめとする国際的に広く用いられている指標を用いることが望ましい。支持療法は分野によって評価する項目が異なるため、個々の尺度の選択については別途指針を作成する。

6.2　緩和治療の研究におけるエンドポイントと評価尺度

6.2.1　エンドポイントの選択

患者の苦痛を軽減することが目的である緩和治療の臨床試験においては、患者の苦痛をエンドポイントとして設定する。例えば、鎮痛薬では痛みの程度、制吐薬では悪心・嘔吐の程度をエンドポイントとする。

苦痛の程度を何によって測定するのが最も適切かは明確にされていない場合が多い。例えば、痛みでは、24 時間の最大の痛み（worst pain）、平均的な痛み（average pain）などがエンドポイントとなりうる。[26] 悪心・嘔吐では、24 時間の最大の悪心（worst nausea）、平均的な悪心（average nausea）、嘔吐回数などがエンドポイントになりうる。このように治療対象となる苦痛は明確であっても、苦痛の評価方法は複数ある場合が多い。将来的には患者にとって最も重要なエンドポイントが個々の苦痛について明確にされることが望ましいが、現状として国際的にも統一されていない。

したがって、現在のところ、先行研究を注意深く参考にして患者にとって重要と考えられるエンドポイントを主要評価項目と設定して、相対的に重要性の低いものを副次評価項

目として設定することが合理的である。同等に重要だと考えられるエンドポイントが複数ある場合にはそれらを複数の主要評価項目（コプライマリーエンドポイント）として設定する[27]。

6.2.2　主要評価項目と副次評価項目の違い

複数の主要評価項目を置いた場合、結果に不一致が生じた場合にどのように解釈するのかという問題が生じうる。そのため、重要であると考えられるエンドポイントについては主要評価項目とし、不一致が生じた場合の対応を決めておく。

主要評価項目と副次評価項目とで結果に不一致が生じた場合、試験治療は患者にとって部分的に有効な可能性があると解釈されうる場合はある。例えば、嘔吐が全くなかった割合は変わらなくても、悪心の強さや嘔吐ののべ回数が減少したならば、治療は部分的に有効な可能性があると解釈して、不利益が小さく他に利用可能な治療がないならば実際の臨床現場として治療を実施することはありうる。ただし、副次評価項目での結果の信頼性は主要評価項目のそれと比べて劣ることを理解し、将来的には同項目を主要評価項目とした臨床試験で有効性の検証を行うことが望ましい。

6.2.3　苦痛緩和における臨床的に意味のある差（Minimum important difference：MID）

臨床試験は、これだけ苦痛が緩和されれば患者にとって意味があると実感できる差、すなわち MID を検出できるようにデザインされる必要がある。

評価尺度で連続変数として扱うことが通例であるもの（QOL 尺度など）は、平均値の差で検討されることが妥当であるが、解釈しやすいという点から、有効率の差も評価項目に設定することを考慮する（例えば、主要評価項目を「NRS の平均値」とした場合に、副次評価項目に「NRS が 2 点以上下がった患者の割合」を加える、など）。

MID は現在ほとんどの苦痛について、定まっていないので、感度分析*として異なるカットオフ値でも有効率を算出することを推奨する（例えば、主要評価項目は「最大の痛みが 33%低下した患者の割合の差」であっても、副次評価項目として「25%低下した患者の割合」「50%低下した患者の割合」を加える、など）。

---

*脚注　感度分析とは情報の不確実性や変動性が、アウトプット（発症確率）の値にどのような影響をもたらすかを把握する手法であり、アウトプットをより適切に認識し、適切な評価を導くことを目的として実施するものである。これによって結論の確かさが増す。

6.2.4　評価尺度

苦痛の評価に当たっては、信頼性と妥当性の確認された症状評価尺度の日本語版を使用する。頻用される Edmonton Symptom Assessment Schedule（ESAS）[28]や Brief Pain Inventory（BPI）[29]をはじめとした症状評価尺度の日本語版が利用可能である。

7. 対象患者の死亡に関する取り扱い

7.1 背景

臨床研究実施中に頻発する重篤な有害事象（Serious Adverse Event: SAE）を適切に評価することは、安全性と効率性の両面において重要である。SAE はまれな事象であることが前提とされているため、SAE が実施医療機関にて発生した場合には、倫理委員会や独立データモニタリング委員会等に、速やかかつ詳細に報告を行うことが求められる。[30]

一方、支持療法・緩和治療、特に緩和治療の臨床研究においては、被験者は原病の悪化とともに状態が悪化するため、研究の実施中に SAE（死亡例）が多数発生することが想定される。研究治療とは無関係の原病の悪化による死亡に関する SAE を頻繁に報告・評価することは、臨床研究の実施に影響を及ぼすだけではなく、真に重要な SAE を見逃す可能性も生じうる。

7.2 安全性情報の管理

適用される規制要件、研究デザインなどに応じ、安全性情報を適切に管理することがまずは重要である。その上で、原病の悪化による死亡が発生することが予測される場合には、あらかじめ、個別のプロトコールで緊急報告の対象外と定めることとする。緊急報告の対象外とする有害事象の状態や事象は試験ごとに検討し、プロトコールに明記する。緊急報告の対象外と規定された以外の SAE が発生した場合には規定の期日内に報告を行う。

原病の悪化による死亡をプロトコールで緊急報告の対象外と定める場合、施設研究責任者により次の要件をすべて満たされていることを必須とする。

- 死亡が予期されるものであったかの評価
- 死亡が原病の悪化によるものであることの確定
- カルテ等の診療文書の確認

すなわち、死亡が予期されるものであり、かつ、明らかに原病の悪化によるものであり、かつ、カルテ等の診療文書に状況が明確に記録されていることが確認された場合に限り、緊急報告の対象外となる。緊急報告の対象外とされた原病の悪化による死亡は、CRF で情報を収集し、データセンターが独立データモニタリング委員会に報告して審査を受ける。

7.3 試験デザイン

被験者の脱落を考慮したサンプルサイズ設計などの研究デザインの考慮を行う。解析においても、患者の脱落等に結論が影響を受けにくい解析方法、あるいは、脱落理由（データの欠測理由）を十分考慮した解析方法の適用が望まれる。特に死亡患者の取り扱いについては、通常の欠測データ解析の考え方とは異なるアプローチが必要であり、解析対象集団の選択も含めて注意を要する。すなわち、通常の欠測データ解析においては、もしデータが測定できていたらどのようなデータが得られていたであろう、という観点から推測を行うが、実際には患者は死亡しておりそのような前提が成り立たないことから、例えば、

推測の対象を「死亡しないであろう患者」とするなど（主要層ストラテジーと呼ばれる）、推測の対象となる集団を考慮した対応が必要となる。[31]

8.　実施体制と品質マネジメント

8.1　基本的な考え方

支持療法・緩和治療の臨床研究に限定されないが、目標とする品質水準は研究によって異なる。適用される規制要件、研究デザインなどに応じ、コストや時間、実現可能性などを考慮しなければならない。被験者の保護、質の確保に加え、「効率」、「費用対効果」を意識し、実施体制を含む研究の品質マネジメントシステムを構築する。実際には、研究事務局に加えて、臨床研究支援組織であるデータセンターあるいは相当するデータマネジャー、生物統計家、（必要に応じて）モニターなどが研究に参加することが望ましい。研究組織によって研究方法は変わりうるので、柔軟で実現可能な対応が必要である。

また、支持・緩和治療の試験は多科、多職種、多施設による試験が多く、試験計画、遂行に対する貢献度に応じて配分される authorship については、研究協力者の間での合意を前提に試験開始前にルールを決定しておくことを奨める。

8.2　研究の品質マネジメントとシステム（Quality Management System (QMS)）の構築[32]

「顧客の要求にあった品質の製品やサービスを経済的に作り出すための一連のプロセス」のことを品質マネジメントと呼び、主要な活動が以下で述べる品質管理・品質保証である。[33]

顧客とは、製品やサービスを受け取る組織または人であり、臨床研究の枠組みでは、もちろん最終的には患者であるが、段階や状況に応じて、研究者、製薬企業、規制当局等多岐にわたる。

品質とは「製品やサービスに備わっている特性の集まりが、要求事項を満たす程度」のことであり、臨床研究にあてはめれば、顧客満足度とは顧客による品質評価、そこでの要求水準とは GCP などの規制要件、人を対象とする医学系研究に関する倫理指針等を満たす性質である。

例えば、製薬企業主導の治験であれば、当座の顧客は、PMDA、厚生労働省であり、そこでの審査に適合し承認されるような治験、すなわち、GCP などに従う試験を実施すればそれは質が高い試験と考えることができる。また、臨床研究者にとって結果の公表を行う媒体として学術雑誌に論文を掲載して公開することが責務であるが、掲載を目指す雑誌がどのような臨床研究の実施を求めているのか、その要件を満たすにはどのような研究が質の高いのかなどが重要な点となる。研究の出口を見据え目的を踏まえた品質水準を計画時に設定する必要がある。品質を担保するうえではプロセス管理の実践が重要であり、研究者間での情報共有と連携が鍵となる。

8.3 研究の品質管理（Quality Control; QC）

品質管理とは、計画段階からデータやプロセスの妥当性、信頼性を確保する手続きを考慮、実行し、実施段階で正しく実行されているかどうかをチェックし、正しく行われていない場合にはデータの修正やプロセスの見直しを行う活動である。重要な品質管理活動を以下に述べる。これらの適切な品質管理を行うためには、研究開始前に品質管理のシステムを構築する、すなわち、作業の標準化（標準業務手順書 Standard Operating Procedure (SOP)、マニュアルなど）を行い、それを適切に運用し、研究の質向上のため、確認とフィードバックを行い、正しく行われていない場合プロセスの修正と改善をそれぞれの業務単位でチェックする必要がある。いわゆる PDCA（Plan：計画、Do：実施、Check：評価、Act：処置）活動に基づき、継続的な改善活動（スパイラルアップ）が重要である。

8.3.1 研究計画書（プロトコール）の作成

プロトコールの完成度を高め、研究の科学性と倫理性を担保する必要がある。これには、プロトコールマニュアルによる標準化、研究事務局とデータセンターとの協力、第三者あるいはプロトコール審査委員会によるレビューなどが有用である。

8.3.2 登録と割り付け（ランダム化）

不適格例や誤登録を防止するため、また、割り付け結果の隠匿を確保するため、統計家によって適切な方法と手順にもとづく割り付けを行う必要がある。研究者とは独立したデータセンター（中央登録センター）の設置や Web-based の登録システムの利用などが有用である。

8.3.3 データの測定、評価

担当医による評価や報告のバイアスを回避する、あるいは、施設間、担当医間のバラツキを小さくするための対処が必要である。薬剤や治療法のマスク化、エラーの少ない標準化された評価項目の使用、測定方法の統一、訓練、研究開始前の研修、事前演習、中央一括測定、中央第三者評価判定、データセンターによるデータマネジメントと中央モニタリングなどが有用である。主要評価項目などに欠測が生じないよう十分予防策を立てるとともに、欠測が生じた場合にはその理由や欠測の原因となったデータやプロセスの情報を収集しておくことは、それらの情報を解析で考慮できる可能性があり有用である。

8.3.4 研究の進捗管理、モニタリング

研究がプロトコールや各種手順書などに従って各施設で適切に実施されているかを確認する。研究参加者の権利と安全性が確保されているか、データが正しく報告されているか、実施されていることがきちんと記録されているか、などについて考慮し、必要に応じて各種文書の改訂やプロセスの改善を考慮する、施設への改善を求める。研究者の自己点検、データセンター（あるいは研究事務局）による進捗管理と中央モニタリング、施設モニタリング、独立データモニタリング委員会（安全性の担保、判断の客観性）の設置などが行われる。研究者へのフィードバックについては、そのリスクに応じたレベル分けが重要であり、ネットワークレベル（あらゆる研究に共通するリスク）なのか、プロトコール

レベル（研究特有で施設によらず想定されるリスク）なのか、あるいは、施設（研究者）レベル（施設（研究者）ごとに想定されるリスク）なのか考慮すべきである。

8.3.5　データマネジメント

データの正確性、完全性を保証し、公正かつ科学的評価が可能なデータの品質水準を担保することが重要である。研究実施者とは独立したデータセンターの設置、妥当性の確認のとれた（バリデートされた）データ管理システムの利用などが有用である。データマネジメントとは、正確でばらつきの少ない質の高いデータを効率的に収集・管理し、データが公正に評価され正しい結論を導くための方法論である。研究計画書（プロトコール）の作成、調査票の作成、データ収集、データチェック、研究者への問い合わせ、研究の進捗管理、データ入力、データクリーニング、解析のためのデータセットの作成など、臨床研究のほぼすべてのプロセスに置いて重要な役割を担っている。臨床研究の質を左右する「データ」をマネジメントする役割であり、単なるデータ収集、入力、チェックだけの役割ではないことを特記する。

8.3.6　臨床研究現場でのデータ収集プロセスの管理

施設（研究者）でのデータの質管理として、ALCOA の原則（Attributable：記録の責任が明確、Legible：判読可能、Contemporaneous：データ発生後、速やかに記録・処理、Original：原本（写しでない）、Accurate：正確）にもとづいたデータ収集が重要である。また、研究者および協力者間での役割分担や情報共有手順など、事前に施設内での実施体制について確認する事も重要となる。

8.3.7　統計解析

解析プロセスの妥当性を担保する必要がある。事前に解析計画を規定（解析対象集団、データの取り扱いにも留意）し、認証（バリデート）された解析パッケージを用いて、可能であればダブルプログラミングなどで結果の再現性を担保する。

8.4　研究の品質保証（Quality Assurance; QA）

研究の品質管理活動は、研究者が主導となって行われるものであり、必ずしも顧客のニーズにあったものではない可能性がある。顧客の視点から、該当研究がある一定水準以上の品質を確保していることの評価を行うことを品質保証という。研究の実施の際に残された「記録（証拠）」を第三者の立場から点検し、データとプロセスの観点から適切に研究が実施されているかどうか確認する。研究全体の質の評価、すなわち、研究が正しく行われたかを評価・判定、結果がでるまでのプロセス全体を保証する。品質保証のための中心的な活動が監査である。

（参考文献）

1. Hui D. Definition of supportive care: does the semantic matter? Curr Opin Oncol. 2014;26(4):372-9.
2. U.S. National Institutes of Health. NCI Dictionary of Cancer Terms [Available from: https://www.cancer.gov/publications/dictionaries/cancer-terms.]
3. World Health Organization. WHO Definition of Palliative Care [Available from: http://www.who.int/cancer/palliative/definition/en/.]
4. Randomized, double-blind, placebo-controlled study to assess the efficacy and toxicity of subcutaneous ketamine in the management of cancer pain. Hardy J, Quinn S, Fazekas B, Plummer J, Eckermann S, Agar M, Spruyt O, Rowett D, Currow DC. J Clin Oncol. 2012 Oct 10;30(29):3611-7. doi: 10.1200/JCO.2012.42.1081. Epub 2012 Sep 10.
5. *Caraceni A. J Clin Oncol. 2004;22:2909-17.*
6. Fentanyl Sublingual Tablets Versus Subcutaneous Morphine for the Management of Severe Cancer Pain Episodes in Patients Receiving Opioid Treatment: A Double-Blind, Randomized, Noninferiority Trial. Zecca E, Brunelli C, Centurioni F, Manzoni A, Pigni A, Caraceni A. J Clin Oncol. 2017 Mar;35(7):759-765. doi: 10.1200/JCO.2016.69.9504. Epub 2017 Jan 23.
7. Treede RD, Jensen TS, Campbell JN, Cruccu G, Dostrovsky JO, Griffin JW, et al. Neuropathic pain: redefinition and a grading system for clinical and research purposes. Neurology. 2008;70(18):1630-5.
8. American Psychiatric Association. Diagnostic and Statistical Manual of Mental Disorders. 5th edition: Amer Psychiatric Pub Inc; 2013.
9. Hui D. Prognostication of Survival in Patients With Advanced Cancer: Predicting the Unpredictable? Cancer Control. 2015;22(4):489-97.
10. Oken MM, Creech RH, Tormey DC, Horton J, Davis TE, McFadden ET, et al. Toxicity and response criteria of the Eastern Cooperative Oncology Group. Am J Clin Oncol. 1982;5(6):649-55.
11. Karnofsky DA, Burchenal JH. The clinical evaluation of chemotherapeutic agents in cancer, in Macleod CM (ed): Evaluation of Chemotherapeutic Agents. New York: Columbia University Press; 1949.
12. Maltoni M, Nanni O, Pirovano M, Scarpi E, Indelli M, Martini C, et al. Successful validation of the palliative prognostic score in terminally ill cancer patients. Italian Multicenter Study Group on Palliative Care. Journal of pain and symptom management. 1999;17(4):240-7.

13. Morita T, Tsunoda J, Inoue S, Chihara S. The Palliative Prognostic Index: a scoring system for survival prediction of terminally ill cancer patients. Supportive care in cancer : official journal of the Multinational Association of Supportive Care in Cancer. 1999;7(3):128-33.
14. Gwilliam B, Keeley V, Todd C, Gittins M, Roberts C, Kelly L, et al. Development of prognosis in palliative care study (PiPS) predictor models to improve prognostication in advanced cancer: prospective cohort study. BMJ. 2011;343:d4920.
15. Masso M, Allingham SF, Banfield M, Johnson CE, Pidgeon T, Yates P, et al. Palliative Care Phase: inter-rater reliability and acceptability in a national study. Palliat Med. 2015;29(1):22-30.
16. Folstein MF, Folstein SE, McHugh PR. "Mini-mental state". Journal of Psychiatric Research. 1975;12(3):189-98.
17. Loudon K, Treweek S, Sullivan F, Donnan P, Thorpe KE, Zwarenstein M. The PRECIS-2 tool: designing trials that are fit for purpose. BMJ. 2015;350:h2147. PMID: 25956159.
18. Ross C. Brownson, Graham A. Colditz and Enola K. Proctor. Dissemination and Implementation Research in Health: Translating Science to Practice. Oxford University Press.
19. Fentanyl Sublingual Tablets Versus Subcutaneous Morphine for the Management of Severe Cancer Pain Episodes in Patients Receiving Opioid Treatment: A Double-Blind, Randomized, Noninferiority Trial. Zecca E, Brunelli C, Centurioni F, Manzoni A, Pigni A, Caraceni A. J Clin Oncol. 2017 Mar;35(7):759-765. doi: 10.1200/JCO.2016.69.9504. Epub 2017 Jan 23.
20. Effect of continuous deep sedation on survival in patients with advanced cancer (J-Proval): a propensity score-weighted analysis of a prospective cohort study. Maeda I, Morita T, Yamaguchi T, Inoue S, Ikenaga M, Matsumoto Y, Sekine R, Yamaguchi T, Hirohashi T, Tajima T, Tatara R, Watanabe H, Otani H, Takigawa C, Matsuda Y, Nagaoka H, Mori M, Tei Y, Kikuchi A, Baba M, Kinoshita H. Lancet Oncol. 2016 Jan;17(1):115-22. doi: 10.1016/S1470-2045(15)00401-5. Epub 2015 Nov 29.
21. Parenteral hydration in patients with advanced cancer: a multicenter, double-blind, placebo-controlled randomized trial. Bruera E, Hui D, Dalal S, Torres-Vigil I, Trumble J, Roosth J, Krauter S, Strickland C, Unger K, Palmer JL, Allo J, Frisbee-Hume S, Tarleton K. Ann Oncol. 2005 Apr;16(4):640-7. Epub 2005 Jan 31.

22. Association between hydration volume and symptoms in terminally ill cancer patients with abdominal malignancies.　Morita T1, Hyodo I, Yoshimi T, Ikenaga M, Tamura Y, Yoshizawa A, Shimada A, Akechi T, Miyashita M, Adachi I; Japan Palliative Oncology Study Group.　J Clin Oncol. 2013 Jan 1;31(1):111-8. doi: 10.1200/JCO.2012.44.6518. Epub 2012 Nov 19.
23. Liverpool Care Pathway for patients with cancer in hospital: a cluster randomised trial.Costantini M, Romoli V, Leo SD, Beccaro M, Bono L, Pilastri P, Miccinesi G, Valenti D, Peruselli C, Bulli F, Franceschini C, Grubich S, Brunelli C, Martini C, Pellegrini F, Higginson IJ; Liverpool Care Pathway Italian Cluster Trial Study Group.　Lancet. 2014 Jan 18;383(9913):226-37.
24. Atropine, hyoscine butylbromide, or scopolamine are equally effective for the treatment of death rattle in terminal care.　Wildiers H1, Dhaenekint C, Demeulenaere P, Clement PM, Desmet M, Van Nuffelen R, Gielen J, Van Droogenbroeck E, Geurs F, Lobelle JP, Menten J; Flemish Federation of Palliative Care. J Pain Symptom Manage. 2009 Jul;38(1):124-33.
25. U.S. NCI Division of Cancer Treatment and Diagnosis. Common Terminology Criteria for Adverse Events (CTCAE) Version 4.0 2009 [updated May 28. Available from: https://ctep.cancer.gov/protocoldevelopment/electronic_applications/ctc.htm.]
26. U.S. Food and Drug Administration. Guidance for industry analgesic indications: developing drug and biological products. 2014.
27. U.S. Food and Drug Administration. Guidance for industry: patient-reported outcome measures: use in medical product development to support labeling claims. Federal Register2009. p. 65132-3.
28. Bruera E, Kuehn N, Miller MJ, Selmser P, Macmillan K. The Edmonton Symptom Assessment System (ESAS): a simple method for the assessment of palliative care patients. J Palliat Care. 1991;7(2):6-9.
29. Cleeland CS1 RK. Pain assessment: global use of the Brief Pain Inventory. Ann Acad Med Singapore. 1994;23(2):129-38.
30. 文部科学省,厚生労働省．人を対象とする医学系研究に関する倫理指針.
31. Addendum to Statistical Principles for Clinical Trials on Choosing Appropriate Estimands and Defining Sensitivity Analyses in Clinical Trials (https://www.pmda.go.jp/int-activities/int-harmony/ich/0031.html) 2)
32. International Council for Harmonisation of Technical Requirements for Pharmaceuticals for Human Use. INTEGRATED ADDENDUM TO ICH E6(R1): GUIDELINE FOR GOOD CLINICAL PRACTICE E6(R2). 2016.
33. INTEGRATED ADDENDUM TO ICH E6(R1): GUIDELINE FOR GOOD

CLINICAL PRACTICE E6(R2) (https://www.pmda.go.jp/int-activities/int-harmony/ich/0028.html)
