# Supplementary material for: Establishment of a research policy for supportive and palliative care in Japan
Source: Jpn J Clin Oncol. 2021 Feb 10;51(4):538–43. doi: 10.1093/jjco/hyab008 (PMC8129624; doi:10.1093/jjco/hyab008)
Supplement: 20200206_ver1_1_hyab008 [file 20200206_ver1_1_hyab008.pdf]

National Research and Development Agency, Japan Agency for Medical Research and Development (AMED)
Practical Research for Innovative Cancer Control, Program No. 18ck0106376h0002
Research for methodology establishment for supportive and palliative care studies

Research Policy in the Areas of Supportive and Palliative Care (General Issues)

Version 1.0 **December 3, 2018 (Japanese ver.)**
English Ver. **July 5, 2019**
Version 1.1 **February 6, 2020**

Authors: Working Group on the Development of Research Policy in the Areas of Supportive and Palliative Care, General Issues

Sadamoto Zenda (Supportive care)
Chief Physician, Department of Radiation Oncology,
National Research and Development Agency National Cancer Center Hospital East

Tatsuya Morita (Palliative care)
Division Director and Deputy Hospital Director,
Palliative and Supportive Care Division,
Seirei Mikatahara General Hospital

Akira Inoue (Supportive care/Palliative care)
Professor, Division of Palliative Medicine,
Tohoku University Graduate School of Medicine

Takuhiro Yamaguchi (Supportive care/Palliative care)
Professor, Division of Biostatistics,
Tohoku University Graduate School of Medicine

Yosuke Uchitomi (Supervising editor)
Director, Supportive Care Development Center,
National Cancer Center Hospital

Research collaborators
Yutaka Matsuoka
Division Chief, Division of Health Care Research,
Center for Public Health Sciences,
National Research and Development Agency National Cancer Center Japan

Takako Nakajima
Professor, Department of Clinical Oncology,
St. Marianna University School of Medicine

Fumio Nagashima
Professor, Department of Medical Oncology,
Kyorin University Faculty of Medicine

Hiroyuki Terakado
Department Director, Department of Pharmacy,
National Research and Development Agency National Center for Global Health and Medicine Hospital

Masakazu Yamaguchi
Department Director, Department of Pharmacy,
National Cancer Center Hospital

Hironobu Hashimoto
Deputy Department Director, Department of Pharmacy,
National Cancer Center Hospital

Masato Yonemura
Member, Safety Management Division, Research Management Section, Clinical Research Support Office,
National Research and Development Agency National Cancer Center Hospital East

Toshikatsu Kawasaki
Department Director, Department of Pharmacy,
National Research and Development Agency National Cancer Center Hospital East

Masakazu Abe
Chief Physician, Division of Gynecology,
Shizuoka Cancer Center

Tomoya Yokota

Chief Physician, Division of Gastrointestinal Oncology,
Shizuoka Cancer Center

Yoshihisa Matsumoto
Chief Physician, Department of Palliative Medicine,
National Research and Development Agency National Cancer Center Hospital East

Miki Kawahara
Assistant Professor, Gerontological Nursing, School of Nursing,
Tokyo Women's Medical University

Akiko Hanai
Contract Researcher, Division of Health Care Research, Center for Public Health Sciences,
National Research and Development Agency National Cancer Center Japan

Sanae Shimada
Contract Research Assistant, Department of Radiation Oncology,
National Research and Development Agency National Cancer Center Hospital East

Takashi Kawaguchi
Assistant Professor, Department of Practical Pharmacy,
Tokyo University of Pharmacy and Life Sciences

Tempei Miyaji
Contract Researcher, Division of Health Care Research, Center for Public Health Sciences, National Research and Development Agency National Cancer Center Japan
Project Assistant Professor, Department of Clinical Trial Data Management,
The University of Tokyo Graduate School of Medicine

Taichi Shimazu

Director, Division of Prevention, Center for Public Health Sciences,
National Research and Development Agency National Cancer Center Japan

Maiko Fujimori
Director, Division of Health Care Research, Center for Public Health Sciences,
National Research and Development Agency National Cancer Center Japan

Collaborative Medical Societies and Respective Review Personnel

Yuko Kitagawa, Chairman, Japan Society of Clinical Oncology
Masakazu Toi, Department of Breast Surgery, Kyoto University Hospital

Kazuo Tamura, President, Japanese Association of Supportive Care in Cancer
Atsushi Sato, Department of Medical Oncology, Hirosaki University Graduate School of Medicine

Toyofumi Hosokawa, President, Japanese Society for Palliative Medicine
Etsuko Aruga, Department of Palliative Medicine, Teikyo University School of Medicine

Tatsuo Akechi, President, Japan Psycho-Oncology Society
Toru Okuyama, Division of Palliative Care and Psycho-oncology, Nagoya City University Hospital

Chiemi Onishi, President, Japanese Society of Cancer Nursing
Kiyoko Kanda, Department of Nursing, Gunma University Graduate School of Health Sciences

Hironobu Minami, President, Japanese Society of Medical Oncology
Ikuo Sekine, Department of Medical Oncology, Tsukuba University Faculty of Medicine

Naoyuki Shigematsu, President, Japanese Society for Therapeutic Radiation

Oncology
Hidefumi Aoyama, Department of Radiology and Radiation Oncology, Niigata University Medical and Dental Hospital

Hitoshi Nakagama, President, Japanese Cancer Association
Yasuhito Uezono, Department of Cancer Pathophysiology

(in random order, honorifics omitted)

Table of Contents

1. Objective of the research policy in the areas of supportive and palliative care
2. Definitions of supportive care and palliative care
   2.1 Background
   2.2 Cancer treatment
   2.3 Supportive care (Supportive care for toxicities arising from cancer treatment)
   2.4 Palliative care (Palliative care for symptoms arising from cancer)
3. Characteristics of supportive and palliative care research
   3.1 Characteristics of supportive care research
      3.1.1 Relationship between cancer treatment and supportive care
      3.1.2 Cooperation with the department providing cancer treatment
      3.1.3 Quality of interventions
         3.1.3.1 Pharmacotherapeutic interventions
         3.1.3.2 Non-pharmacotherapeutic interventions
      3.1.4 Adverse events and adverse reactions
   3.2 Characteristics of palliative care research
      3.2.1 Absence of established standard of care
      3.2.2 Flexibility of protocol treatment
      3.2.3 General condition of participating patients
      3.2.4 Location of treatment for patients
      3.2.5 Informed consent
4. Target population for research
   4.1 Systematic differences between the study population and the patient population in clinical practice
   4.2 Target population for supportive care research
      4.2.1 Broader eligibility of the target population for cancer treatment
   4.3 Target population for palliative care research
      4.3.1 Definition of target population in palliative care
      4.3.2 Balance between homogeneity in the study population and feasibility
      4.3.3 Age restrictions
      4.3.4 General condition restrictions
      4.3.5 Clinician prediction of survival (CPS)
      4.3.6 Cognitive function
      4.3.7 Definition of advanced cancer patients
      4.3.8 Laboratory test data

5. Research design
  5.1 Confirmatory and exploratory studies
  5.2 Supportive care research designs
    5.2.1 Research objectives
    5.2.2 Confirmatory study designs
      5.2.2.1 Placebo-controlled, double-blind, randomized studies
        5.2.2.1.2 Active-controlled, double-blind, randomized studies
      5.2.2.2 Open-label, randomized studies
        5.2.2.2.1 Endpoint-blinded, open-label, randomized studies
        5.2.2.2.2 Open-label, randomized studies using objective measures such as survival
      5.2.2.3 Single-arm confirmatory studies
    5.2.3 Exploratory research designs
      5.2.3.1 Exploratory study in preparation for a confirmatory study
    5.2.4 Explanatory trials and pragmatic trials
    5.2.5 Dissemination and implementation research
    5.2.6 Observational research
  5.3 Palliative care research designs
    5.3.1 Research objectives
    5.3.2 Palliative care research designs
      5.3.2.1 Confirmatory studies
        5.3.2.1.1 Double-blind, randomized studies
          5.3.2.1.1.1 Placebo-controlled, double-blind, randomized studies
          5.3.2.1.1.2 Active-controlled, double-blind, randomized studies
        5.3.2.1.2 Single-arm confirmatory studies
        5.3.2.1.3 Cluster-randomized studies
        5.3.2.1.4 Confirmation of effectiveness in clinical practice (pragmatic studies)
      5.3.2.2 Exploratory research
        5.3.2.2.1 Exploratory study in preparation for a confirmatory study
        5.3.2.2.2 Exploratory randomized studies
        5.3.2.2.3 Single-arm studies and observational studies
6. Endpoints and assessment measures
  6.1 Endpoints and assessment measures in supportive care research
    6.1.1 Primary endpoints
      6.1.1.1 Supportive care for acute toxicity affecting the outcome of cancer treatment
      6.1.1.2 Supportive care for acute adverse events or late adverse events in cancer treatment affecting QOL

6.1.2 Other endpoints
6.1.2.1 Assurance of no interference with the effects of cancer treatment
6.1.3 Outcome measures
6.2 Endpoints and outcome measures in palliative care research
6.2.1 Selection of endpoints
6.2.2 Difference between primary endpoints and secondary endpoints
6.2.3 Clinically meaningful difference (minimal important difference: MID) in reduction of pain or symptoms
6.2.4 Outcome scales
7. Handling of deaths of subjects
7.1 Background
7.2 Management of safety information
7.3 Study designs
8. Operational structure and quality management
8.1 Basic principals
8.2 Quality management of research and establishment of a quality management system (QMS)[30]
8.3 Quality control (QC) of research
8.3.1 Research protocol preparation
8.3.2 Enrolment and randomization
8.3.3 Data measurement and assessments
8.3.4 Research progress management and monitoring
8.3.5 Data management
8.3.6 Data collection process management at each study site
8.3.7 Statistical analysis
8.4 Research quality assurance (QA)
(References)

1. Objective of the research policy in the areas of supportive and palliative care

The objective of this policy is to produce a set of guidelines for implementation of clinical research, particularly clinical studies, in supportive care and palliative care. This policy includes the following components of clinical research: (1) definitions of supportive care and palliative care, (2) research characteristics, (3) target population, (4) design, (5) endpoints and assessment measures, (6) handling of deaths of participating patients, and (7) administrative structure and quality management.

Given that different specialty areas have different ideals regarding supportive and palliative care research, this policy provides a basic common framework that can be shared across different specialty areas. This policy primarily focuses on pharmacotherapies and medical interventions, but may be used as a reference for the planning of clinical studies on non-invasive or minimally invasive interventions, including certain psycho-behavioral interventions, nursing interventions, or rehabilitation. This document aims to establish the basic policy for clinical research in supportive and palliative care, and thus does not apply to clinical research/studies/trials regulated by the Clinical Trials Act; i.e., clinical research/studies/trials of unapproved or off-label drugs/devices conducted under the Pharmaceuticals, Medical Devices and Other Therapeutic Products Act (PMD Act) and funded by drug/device companies.

As the original version of this policy was written in Japanese and in line with the circumstances in Japan, matters related to unusual Japan-specific circumstances were modified for a non-Japanese audience in this English version.

2. Definitions of supportive care and palliative care

The definitions of supportive care and palliative care are provided below, along with their history and background.

2.1 Background

In recent years, the importance of not only treating cancer but also relieving pain and discomfort and providing psychosocial support for patients suffering from the disease has been increasingly emphasized in cancer care. Terms such as supportive care (supportive treatment) and palliative care (palliative treatment) have been used to collectively refer to various methods of support other than treatment of the disease itself (i.e., cancer treatment). However, these terms do not have clear international definitions, nor is there a consensus on the Japanese translation. Based on these circumstances, this document sets the minimally required definitions of supportive care and palliative

care for the purpose of yielding high-quality evidence from implementation of clinical research (studies).

Supportive care originally referred to treatment in oncology settings to prevent or reduce cancer treatment-related complications (e.g., antiemesis, infection control). Recently, however, the term "supportive care" has been used to broadly refer to treatments and methods of support provided to improve the quality of life of patients. The National Cancer Institute in the United States (as of November 2017) uses the term supportive care as a synonym for palliative care. Supportive care has a comprehensive meaning, and includes treatment of side effects caused by cancer treatment, relief of cancer symptoms, psychological care, provision of information about the disease condition and treatment, treatment of comorbidities, support for family members and caregivers, and care for problems that occur in the terminal phase.[(1)] [(2)]

Palliative care originated from hospice care. Hospices were originally facilities for pilgrims or sick people mainly in Europe in the 11th century. Etymologically, the word "hospice" derives from the Latin word "hospitium" (meaning hospitality). Subsequently in 1967, St Christopher's Hospice was opened, and the modern hospice movement spread across the world. In the 1970s, when hospital care was introduced to Montreal, Canada, the term "palliative care" was first used in place of hospice care because in the French-speaking areas the word hospice referred to a shelter for poor people. Subsequently in 1990, the World Health Organization (WHO) published a report titled "Cancer Pain Relief and Palliative Care", and the term palliative care became internationally accepted. Currently, the WHO defines palliative care as an approach that improves the quality of life of patients and their families facing problems associated with life-threatening illness, through the prevention and relief of suffering by means of early identification and impeccable assessment and treatment of pain and other problems, physical, psychosocial and spiritual (2002).[(3)]

Therefore, the terms "supportive care" and "palliative care" in their broad sense overlap and cannot be differentiated. However, for the purposes of promoting clinical research (studies), in this policy, narrower definitions of the terms would be more desirable for clarifying the core meanings of the terms and thereby the services meant by these terms. Thus, in this policy document, the original meaning of "supportive care" (i.e., prevention or relief of complications associated with cancer treatment) is more appropriate; therefore, "supportive care" is defined as "**supportive care for side effects induced by cancer treatment**". On the other hand, among the palliative care services, those involving pharmacotherapy or invasive treatment are sometimes referred to as "palliative medicine". Given that this policy document focuses on research studies involving medical interventions such as pharmacotherapies, the term "palliative care" is defined as "**palliative care for cancer-induced**

symptoms". These definitions do not negate the existence of other terms currently used to refer to general clinical concepts.

2.2 Cancer treatment

Cancer treatment refers to treatment that directly acts on the tumor to exert anti-tumor effects (disease-modifying effects), and specifically includes surgical treatment, cancer pharmacotherapy, and radiotherapy.

2.3 Supportive care (Supportive care for toxicities arising from cancer treatment)

Supportive care refers to treatment performed for the prevention or symptomatic relief of adverse reactions to cancer treatment. Adverse reactions include post-treatment complications and sequelae.

Examples of supportive care include treatments for chemotherapy-induced nausea and vomiting (CINV), febrile neutropenia (FN), chemotherapy-induced peripheral neuropathy (CIPN), radiation dermatitis, postoperative wound pain, and postoperative delirium.

2.4 Palliative care (Palliative care for symptoms arising from cancer)

Palliative care refers to treatment performed for the prevention or symptomatic relief of cancer-related pain, discomfort, or symptoms.

Examples of palliative care include treatments for cancer pain, malignant bowel obstruction (MBO) due to cancerous peritonitis, cancer-related fatigue (CRF), and cancer-related dyspnea.

3. Characteristics of supportive and palliative care research

While there are many common features between general clinical studies and clinical studies in supportive or palliative care, there are also important differences and characteristics, which are specified below.

3.1 Characteristics of supportive care research

While clinical studies in supportive care are in principle conducted according to the same methodology as that for general clinical studies including clinical studies on cancer treatments, they also have the characteristics described below.

3.1.1 Relationship between cancer treatment and supportive care

Given that supportive care refers to treatment for adverse reactions that occur as a result of cancer

treatment, any study protocol for a novel supportive care strategy is prepared on the assumption that the target cancer treatment would be widely used in clinical practice in the future. If the target cancer treatment is not used in clinical practice, its associated supportive care will not be used. Thus, such a study plan should be carefully prepared.

3.1.2 Cooperation with the department providing cancer treatment

When a clinical study in supportive care is conducted, cancer treatment and supportive care are often provided by different departments. If this is the case, close cooperation with the department providing the cancer treatment is essential for the conduct of the clinical study in supportive care. For example, in a clinical study on the anti-symptomatic effect of a new gargle agent for stomatitis due to head and neck radiotherapy, cancer treatment is provided by the radiology department or otorhinolaryngology department, while supportive care is provided by the dentistry department.

In planning a clinical study in supportive care, the research group should include researchers from the department that will be providing the treatment, and a cooperative relationship should be established so that co-researchers from the department providing cancer treatment will also play a central role in the study. This is important because such cooperation has a great influence on all steps in the clinical study, including the development speed, patient recruitment, and dissemination and implementation of research results.

3.1.3 Quality of interventions

3.1.3.1 Pharmacotherapeutic interventions

For pharmacotherapeutic interventions, the minimally required quality level should be ensured by pre-defining the timing of administration of the drug used for supportive care and the criteria for starting/delaying/stopping treatment to limit researchers' discretion, as with general clinical studies.

3.1.3.2 Non-pharmacotherapeutic interventions

Interventions in supportive care can be non-pharmacotherapeutic. In contrast to pharmacotherapy that is given orally or by intravenous infusion, where administration into the body can be performed without fail, non-pharmacotherapeutic interventions such as nursing care or exercise therapy require involvement of both the patients and medical professionals. Even for non-pharmacotherapeutic interventions, the quality should be maintained above a certain level to ensure reliability of the study results.

Possible methods for securing the quality of non-pharmacotherapeutic interventions include employing a system that only allows participation by intervention providers who are adequately

qualified for the intervention, or pre-study practical training of medical professionals from the participating study centers. When cooperation of patients and/or their family members is required, a certain level of intervention quality should be maintained by preparing detailed written procedures in addition to the protocol and informed consent form.

3.1.4 Adverse events and adverse reactions

An adverse event is defined as any unfavorable and unintended sign (e.g., an abnormal laboratory finding), symptom, or disease temporally associated with the use of a medicinal product, radiotherapy, or surgery, whether or not it is thought to be related to the treatment. An adverse reaction is defined as any adverse event that could be causally related to the treatment, including responses to medicinal products or radiotherapy or complications of surgery, according to the most recent definition in the Common Terminology Criteria for Adverse Events (CTCAE) ver. 3.0 or later.

Research in supportive care is typically conducted for cancer treatment, which is likely to cause serious adverse reactions. Reported adverse events can be adverse reactions to either the cancer treatment or supportive care, for which differentiation in reporting should be stipulated in the protocol.

3.2 Characteristics of palliative care research

Patients receiving palliative care are typically ceasing cancer treatment and vulnerable. When the study population markedly differs from the patient population in clinical practice, the study should be designed with consideration for the surrounding situation (see Figure 1b). Other important issues for clinical research in palliative care are specified below.

3.2.1 Absence of established standard of care

In current palliative care, there is no single recommended treatment for any given type of symptom. This is because, unlike cancer treatment, there is no established standard of care in palliative care for any given type of symptom. Researchers should keep in mind that current research primarily seeks clues to establish a standard of care, while no treatment is currently regarded as the standard of care.

3.2.2 Flexibility of protocol treatment

Characteristically, palliative care in clinical practice is often modified according to the patient's condition because the patient's response to a given palliative care quickly becomes apparent (i.e., in several hours to several days). In contrast, because cancer treatment is ultimately evaluated based on the survival outcome, the efficacy cannot be assessed immediately after the start of treatment but only after months or years. This means that, to reflect treatments in clinical practice, the criteria for dose reduction, delay, and discontinuation of protocol treatment should be flexible. For example, the

dose of analgesic medication should be allowed to be titrated to achieve the necessary analgesia according to the patient’s condition.[(4)(5)]

In addition, in exploratory, proof-of-concept stages of research, studies on interventions that are yet to be fully standardized can be meaningful if they collect information that can guide modifications to daily treatment practices. For example, a study may compare the effectiveness of different analgesics, such as transmucosal fentanyl and injectable morphine, to explore their relative effectiveness.[(6)]

3.2.3 General condition of participating patients

The prognosis of patients tends to be short in palliative care studies. Thus, it is important to: (1) set a study population that would allow completion of the study, (2) assess the study feasibility using a preliminary study, (3) employ the shortest possible study duration, and (4) predefine the handling of expected deaths of patients.

3.2.4 Location of treatment for patients

Potential subjects of clinical studies in palliative care exist not only in particular departments of hospitals but also in other parts of hospitals or in the community outside hospitals. Thus, estimation of the number of potential subjects and the patient accrual rate at a particular study site is very important.

3.2.5 Informed consent

Cognitive impairment commonly coexists with disease progression. Thus, obtaining informed consent from patients can often be impossible. Use of in-advance consent from the patient or consent from the patient’s representative should be carefully considered.

4. Target population for research

In research studies in supportive care and palliative care, consideration of the items below is recommended for better dissemination and implementation of the study results in clinical practice.

4.1 Systematic differences between the study population and the patient population in clinical practice

In both supportive care and palliative care, there can be systematic differences between the study population and patient population in clinical practice.

When the study population is narrow, a study will only enroll patients in better general condition than patients in clinical practice, leading to low generalizability of the study results (Figure 1a). In other words, the study will have low external validity. On the other hand, when the study population is broad due to excessive consideration for generalizability, the baseline characteristics of patients receiving supportive care will be highly variable, leading to the risk of a diluted effect size and difficulty with interpretation of the study results. Additionally, patients receiving palliative care who are in poor general condition may be enrolled, leading to potential difficulty with implementation of the study (Figure 1c). In other words, the study will have low internal validity.

Thus, for clinical studies in supportive care and palliative care, researchers should choose the optimal target population by taking into account the differences between the ideal study population (ideal world) and the actual patient population in clinical practice (real world) (Figure 1b). It is necessary to not only conduct clinical studies for verification, but also to conduct subsequent observational studies or big data investigations of the treatment in the actual patient population in clinical practice (i.e., patients often excluded from clinical studies) to monitor whether the clinical study results are reflected in the treatment of patients in clinical practice.

Figure 1 Clinical study populations for supportive care and palliative care

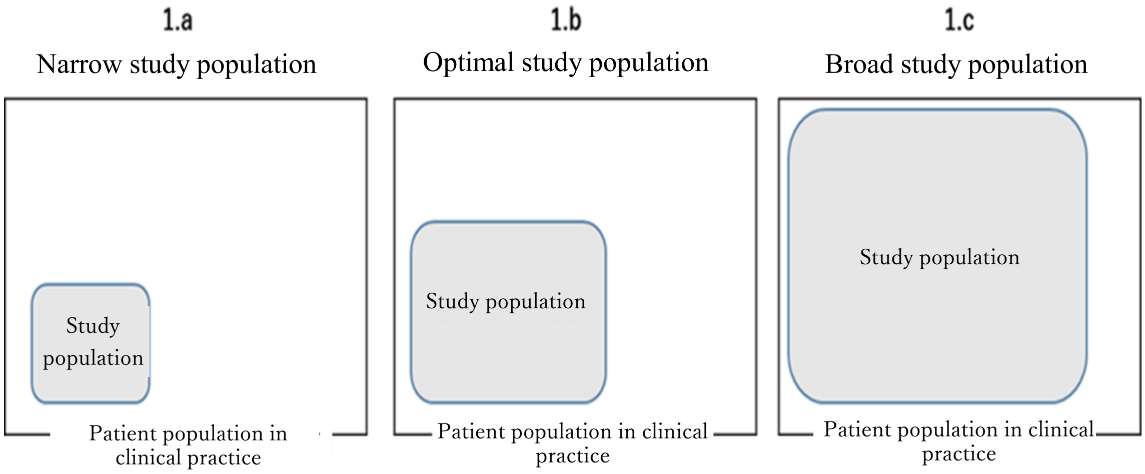

4.2 Target population for supportive care research

4.2.1 Broader eligibility of the target population for cancer treatment

For efficacy studies, efforts should be made to ensure that the background of the study population is as homogeneous as possible to avoid dilution of the effects (Figure 1a). However, after a review, the study results must be adopted in clinical practice as promptly and as broadly as possible. Therefore, to allow for better extrapolability of the study results to the actual patient population in clinical practice, even those with minor differences in cancer treatment (e.g., drugs, procedures), eligibility criteria should not be too restrictive (Figure 1b).

For example, when planning a study on new supportive care strategies for controlling a certain toxicity specific to a drug (Drug A), the eligibility criteria for the disease and medication should not be too restrictive, and should allow the use of Drug A in multiple cancer sites and/or multiple regimens. One good specific example is that a study that aims to demonstrate the benefits of an antiemetic drug as supportive care in moderately or highly emetogenic chemotherapy should not be restricted to a single disease or regimen, but should instead be designed to allow multiple relevant regimens and multiple cancer sites. Similarly in surgery, the eligibility criteria for studies in supportive care should not be too restrictive in terms of the target disease and surgical technique, and should allow broader conditions, such as other surgical techniques with similar invasiveness or other surgical techniques for the same site. When use of broader eligibility criteria is difficult due to the study operation, expansion of the indication (disease, procedure/regimen) as the study exit strategy should be discussed and its process should be documented in the protocol.

Researchers should keep in mind that the ultimate goal of clinical studies in supportive care is “wider

dissemination and better implementation of the obtained findings in clinical practice".

4.3 Target population for palliative care research

4.3.1 Definition of target population in palliative care

In research in palliative care, the diagnostic criteria for the study population may not be clear. Wherever possible, however, the study population should be defined using diagnostic criteria with an international consensus. For example, the diagnostic criteria for neuropathic pain may be based on the definition provided by the International Association for the Study of Pain (IASP)[7], while the diagnostic criteria for depression may be based on the criteria in the Diagnostic and Statistical Manual of Mental Disorders (DSM).[8]

In the absence of diagnostic criteria with an international consensus for defining the study population, the study population should be defined with reference to preceding studies and based on the agreement of specialists involved from multiple areas to ensure objectivity and reliability. Before planning an interventional study, it is desirable to conduct an observational study to examine the reliability and validity of the diagnostic criteria. Given that the definition of the study population greatly affects comparability with preceding studies and study conclusions, it is important that researchers do not use their own definitions.

4.3.2 Balance between homogeneity in the study population and feasibility

In all areas of clinical research, the study population must be as homogeneous as possible to enable application of the research results to individual patients in clinical practice. When the study population is heterogeneous, the effects tend to be diluted or nullified. For example, the study population is more homogeneous when a study only enrolls patients with pancreatic cancer with related visceral pain or neuropathic pain at a specific site, rather than any patients with cancer pain.

However, patient accrual is often impossible for a clinical study that plans to enroll a homogeneous population. Thus, although a study population should be as homogeneous as possible for ideal planning, finding a balance with feasibility is important.

When the study population is heterogeneous, the study should collect information on baseline characteristics to allow for subgroup analysis.

4.3.3 Age restrictions

In research in palliative care, the upper age limit is not an essential factor for defining the study population. This is because in clinical practice, patients receiving palliative care are mostly elderly,

and the calendar age alone is not a determinant of patient vulnerability. Additionally, patients who are inappropriately enrolled would be excluded from the study due to the primary physicians' decision in actual clinical trials. However, setting an upper age limit should be considered when age is anticipated to have a major effect on the intervention or outcome, such as when cognitive function is the primary endpoint of the study.

As for the lower age limit, underage patients require special considerations in the informed consent process, as with other clinical studies.

4.3.4 General condition restrictions

As with age, patients in poor general condition are the primary target population of palliative care research. Thus, subjects should include patients in poor general condition so that the study population reflects real-world patients receiving palliative care. However, a clinical study must exclude patients who are unlikely to survive to the end of the study period. Thus, when a subject's general condition needs to be assessed, the clinician prediction of survival (CPS)(9) should be the standard approach, and expressions such as "patients with life expectancy of at least one month" should be used.

Given that the CPS is subjective in nature, eligibility criteria should include objective and interpretable measures of performance status (ECOG PS(10), KPS(11)) wherever possible.

In addition, information regarding the patient's general condition such as prognostic factors should be collected to clearly describe the study population.

4.3.5 Clinician prediction of survival (CPS)

The CPS is a patient's survival prognosis predicted by the attending physician based on the physician's clinical judgment and experience. The CPS is known to be systematically optimistic. In other words, even with the use of CPS, many patients in a study will have shorter survival than predicted. Thus, a more objective prognostic tool is required to predict the prognosis of study populations in the future. Current objective prognostic tools include the Palliative Prognostic Score (PaP score)(12), Palliative Prognostic Index (PPI)(13), Prognosis in Palliative care Study predictor models (PiPS models)(14), and Palliative care Phase(15), but their use in clinical studies is limited. For the time being, the CPS should be used as the standard measure of general condition concurrently with the Eastern Cooperative Oncology Group (ECOG) performance status score wherever possible.

4.3.6 Cognitive function

In palliative care research involving endpoints that are subjectively assessed by patients, assessment

of the cognitive function of the patients is important, and in principle, the use of tools such as the Mini-Mental State Examination (MMSE)[16] is recommended. However, it would obviously be inappropriate to perform cognitive function testing in critical situations, such as in a study on palliative care in end-of-life dyspnea. Thus, in palliative care research that uses endpoints that are subjectively assessed by patients, patients may be regarded as having a certain level of competency if they are "capable of providing informed consent" based on observation by the researcher.

4.3.7 Definition of advanced cancer patients

To define a patient population in palliative care settings, it is often necessary to define patients with advanced cancer. Previously, the terms "terminally ill cancer patients" and "incurable cancer patients" were used. However, these terms have unclear definitions and are not recommended. In most studies, the study population can be described using the definition "metastatic or locally advanced cancer patients". Cancer types may be specified, such as "clinical stage IV lung cancer".

4.3.8 Laboratory test data

In clinical practice in palliative care, treatment decisions may not be based on laboratory data. However, clinical studies should collect laboratory data on certain parameters (e.g., parameters related to the metabolism of the study drug) to ensure minimally required safety in the study (such as renal function tests for a study drug that is excreted via the kidneys). The test parameters measured in a study should preferably be determined in light of the target patients' conditions and the invasiveness of data collection methods.

5. Research design

This section provides an outline of the research designs used in clinical studies in supportive care or palliative care. Detailed procedures for research methods are provided in separate sections. Although some important points regarding research designs apply to both supportive care and palliative care, this section describes specific important points for supportive care and palliative care under separate headings.

5.1 Confirmatory and exploratory studies

Studies in supportive care and palliative care typically evaluate drugs or dosage regimens that are already in use in clinical practice, and thus the classification of phase I/II/III used in the development of treatments often does not apply, except for new drugs. Thus, studies are classified as confirmatory or exploratory in this section.

Confirmatory studies are studies conducted to establish the usefulness of a treatment. Other studies

are classified as exploratory (Figure 2).

Figure 2. Confirmatory studies and exploratory studies

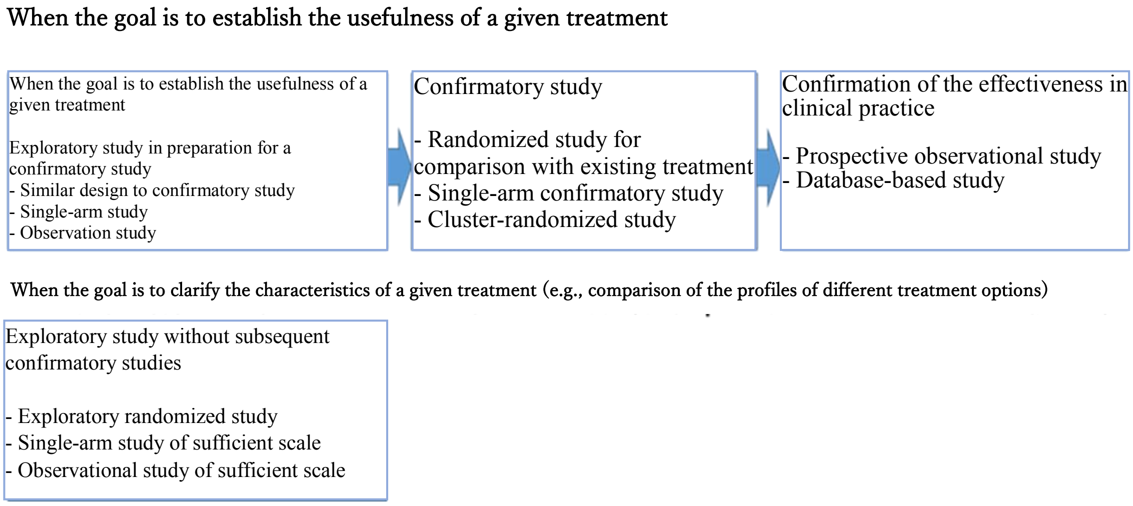

5.2 Supportive care research designs

5.2.1 Research objectives

Supportive care is provided to prevent or reduce symptoms of adverse reactions to cancer treatment, and is consequently expected to maximize the potential of cancer treatment and improve the treatment outcome (i.e., prolongation of survival). At the same time, supportive care can prevent or reduce factors that interfere with activities of daily living during and after cancer treatment (i.e., QOL maintenance and improvement).

Studies in supportive care should therefore employ endpoints that measure the above items, and assess achievement of the above-mentioned objectives.

5.2.2 Confirmatory study designs

To conduct a study in supportive care to determine whether a new intervention or drug treatment (new treatment) would be beneficial in clinical practice, in principle, a randomized confirmatory study should be conducted using no treatment or the current standard of care as a control. Given that objectivity is important in the interpretation of results for supportive care, efforts should be made to maintain objectivity (e.g., use of placebo, central assessment, rater blinding).

5.2.2.1 Placebo-controlled, double-blind, randomized studies

The control group uses a placebo and the study is blinded. The methodologies are the same between supportive care studies and other clinical studies. Determination of the standard of care via double-

blind randomized studies is ideal.

Endpoints may have poor objectivity in supportive care confirmatory studies compared with general oncology clinical studies. For such studies, the use of a double-blind randomized design is strongly recommended to ensure neutrality of the assessments.

5.2.2.1.2 Active-controlled, double-blind, randomized studies

When an existing treatment is considered an established standard of care, a comparative study should be conducted using the standard of care as the control.

When a commonly used treatment exists without consensus as a standard of care, and when researchers are interested in whether the new treatment is more beneficial than the existing treatment, a comparative study should be conducted using the existing treatment as a control. In such instances, in principle, the existing treatment must have demonstrated effectiveness over placebo.

5.2.2.2 Open-label, randomized studies

5.2.2.2.1 Endpoint-blinded, open-label, randomized studies

An open-label design is used for studies in which the control group and the test group are obviously different (e.g., physiotherapy vs. topical agent applied for dermatitis). In open-label clinical studies, endpoint assessments by the researchers who enrolled the patients are typically biased, thus requiring central assessment by blinded raters. In the area of supportive care, the control group may use no intervention due to the absence of a standard intervention for a given symptom. Even in such instances, randomized studies with the control group using no intervention are desirable. If no intervention is unacceptable, studies may be designed to compare the test intervention with the usual intervention (treatment as usual [TAU]) to reflect clinical practice, but such studies would be less likely to detect a difference between the groups and thus require caution.

5.2.2.2.2 Open-label, randomized studies using objective measures such as survival

When the outcome measures are less liable to bias (e.g., overall survival, centrally assessed progression-free survival/relapse-free survival), open-label, randomized studies can be confirmatory.

Even when the primary endpoint is objective, if an important secondary endpoint has a strong subjective element, secondary and lower endpoints should preferably be centrally assessed by blinded raters.

5.2.2.3 Single-arm confirmatory studies

Single-arm studies are in principle not conducted as confirmatory studies. However, only in special circumstances (e.g., when comparison with no treatment is ethically unacceptable, in the absence of alternative interventions), single-arm studies with adequate sample sizes may be confirmatory.

One example is a study investigating whether a new device for a certain procedure is superior (or non-inferior) to a conventional device for the same procedure, where the new device is an improved version of the conventional device and is already marketed and widely used in clinical practice. In such an instance, conducting a randomized study using the old version of the device would be impractical, and thus a single-arm study using appropriate endpoint(s) may be conducted for confirmatory purposes.

5.2.3 Exploratory research designs

5.2.3.1 Exploratory study in preparation for a confirmatory study

An exploratory study conducted in preparation for a confirmatory study aims to collect data necessary for implementation of the subsequent confirmatory study. In addition to safety and efficacy assessments using the same basic methodologies as those for clinical trials, feasibility of the study (e.g., patient accrual ability, protocol compliance rate) should also be assessed.

Supportive care largely involves cooperation with other departments, and many potential institutional barriers may only be revealed after initiation of the study. When a multicenter confirmatory study is planned, an exploratory study should be conducted in advance at a single center or several centers in cooperation with other departments to examine the feasibility. Detailed procedures, researcher training, and post-study compliance assessments should be prepared at this stage.

An exploratory prospective study may be omitted if the hypothesis is supported by existing data from large-scale clinical trials on multiple cancer treatments and sufficient data are also available on adverse events.

5.2.4 Explanatory trials and pragmatic trials

Clinical trials are classified not only as confirmatory or exploratory as mentioned above, but also as explanatory or pragmatic. Explanatory trials aim to evaluate efficacy under ideal conditions, while pragmatic trials aim to evaluate effectiveness in clinical practice. Explanatory trials and pragmatic trials are not clearly separate categories, but are the two ends of a continuum.

Pragmatic trials are characteristically similar to clinical practice in terms of eligibility criteria, patient recruitment efforts, place of intervention (e.g., primary care vs. specialty care), an organization's source/medical professional specialty, method used to deliver the intervention to medical professionals, intervention protocol compliance, and intensity of follow-ups and measurements. Additionally, the primary outcome should be an outcome important to patients and used in clinical practice. Primary analysis should use all data (intention-to-treat analysis).(17)

Given that clinical studies in supportive care are typically conducted at institutions with mature interprofessional collaborations, inter-center differences can be a major problem for generalizability. To address this, a pragmatic trial is recommended for verification of the effects. A pragmatic trial is also recommended after a confirmatory study is conducted only using a single-arm or only at several study centers under good conditions.

5.2.5 Dissemination and implementation research

When a certain intervention is recommended based on efficacy or effectiveness but is not widely used in clinical practice, dissemination and implementation research is conducted to facilitate adoption of the intervention in clinical practice. Such research aims to identify the underlying factors interfering with the dissemination of effective interventions and to promote wide and prolonged use of effective interventions in clinical practice using multiple implementation strategies with approaches for patients, family members, medical professionals, institutional decision makers, environments, and policy.(18)

5.2.6 Observational research

To assess whether obtained knowledge has the appropriate impact on clinical practice, observational research is recommended, such as a prospective observational study at a larger number of study centers. Alternatively, a fact-finding survey on the use of supportive care among society members or a survey using diagnostic procedure combination (DPC) data can be conducted.

5.3 Palliative care research designs

5.3.1 Research objectives

The objective common to palliative care studies is to provide knowledge that contributes to reducing patients' symptoms. Individual studies can be "confirmatory" (i.e., conducted to establish the efficacy of a certain treatment) or "exploratory" (conducted to determine the characteristics of a certain treatment) (Figure 2).

In principle, conducting confirmatory studies to establish efficacy is ideal for all palliative care

interventions. However, in light of the characteristics of palliative care as stated in Section 3.2, confirmatory studies are not the only studies that contribute to clinical practice, and not all confirmatory studies are feasible. Depending on the clinical question, a study “clarifying the characteristics of a certain treatment” may aid the judgment of clinicians. For example, a study may compare the effectiveness of different analgesics, such as transmucosal fentanyl and injectable morphine, to explore the relative effectiveness.(19)

Observational research that aims to better characterize a treatment already in use may in general be of relevance if the characterization will be helpful in clinical practice. In addition, observational research on a research topic in a vulnerable patient population conducted in all patients in the target population, rather than a confirmatory study in a very limited patient population, may be more likely to yield answers to clinical questions. Examples of this include studies on palliative sedation for refractory symptoms and artificial hydration therapy.(20)(21)(22) Use of a research design that allows achievement of the research objective based on clinical questions is important.

5.3.2 Palliative care research designs

5.3.2.1 Confirmatory studies

5.3.2.1.1 Double-blind, randomized studies

Endpoints used in palliative care studies have poor objectivity compared with general oncology clinical studies. Thus, to ensure the neutrality of endpoint assessments, double-blind randomized studies are typically considered necessary.

5.3.2.1.1.1 Placebo-controlled, double-blind, randomized studies

To demonstrate the efficacy of a medication, a confirmatory study should be conducted as a double-blind, placebo-controlled, randomized study. This is because changes in symptoms are affected by various factors, including time, effects of treatment other than the palliative care being tested in the study, effects of treatment on diseases or conditions, effects of psychosocial support, and the placebo effect. When using a placebo, to avoid disadvantaging patients, the study treatment must have no established efficacy, adequate informed consent must be obtained, and rescue measures in the event of symptom exacerbation (e.g., use of rescue medication) must be available. To provide treatment to all patients, possible implementation of a fast-track study (a study that uses randomization according to the timing of intervention) should be considered.

When a given drug has established efficacy sufficient to obtain a drug license in other areas, placebo-controlled studies are not required unless they are conducted to show reproducibility in other specific populations.

5.3.2.1.1.2 Active-controlled, double-blind, randomized studies

When existing treatment is considered an established standard of care, a comparative study should be conducted using the standard of care as the control.

When a commonly used treatment exists without consensus as a standard of care, and when researchers are interested in whether a new treatment is more beneficial than an existing treatment, a comparative study should be conducted using the existing treatment as the control. In such instances, in principle, the existing treatment must have demonstrated effectiveness over placebo.

5.3.2.1.2 Single-arm confirmatory studies

When randomized studies cannot be conducted for certain reasons (e.g., the treatment is already recommended by Japanese guidelines based on non-Japanese findings), well-designed single-arm studies may be conducted for confirmatory purposes. Such studies must enroll a homogeneous group of patients, with appropriate setting of thresholds, sample size and other relevant parameters.

5.3.2.1.3 Cluster-randomized studies

Cluster-randomized studies are studies in which groups (or units or clusters) of patients (e.g., communities, institutions), rather than individual patients, are randomized to different treatments. A cluster-randomized design is a possible option for comparison of therapeutic methods or approaches that are used in groups of patients. An example is a study that investigates the entire effect of a clinical pathway or treatment algorithm for a certain type of symptom.

5.3.2.1.4 Confirmation of effectiveness in clinical practice

In the area of palliative care, patients' conditions in a confirmatory study population are often different from those in the actual patient population in clinical practice (Figure 1). Thus, after completion of a confirmatory study, it is desirable to conduct an adequate-scale prospective observational study or database research to confirm the effectiveness and safety, for which due consideration is warranted from early stages of planning.

5.3.2.2 Exploratory studies

The objective of descriptive studies or exploratory studies can be either to clarify the hypothesis to be tested in a subsequent confirmatory study (hypothesis gathering) or to obtain descriptive results.

5.3.2.2.1 Exploratory study in preparation for a confirmatory study (e.g. a pilot randomized

controlled trial, a feasibility study of a randomized controlled trial)

An exploratory study in preparation for a confirmatory study (Figure 2) aims to collect data necessary for implementation of a confirmatory study. An exploratory study will therefore collect data not only to estimate the effects of a given treatment, but also to determine the feasibility of a planned confirmatory study's design and the appropriateness of the endpoints to be employed. While exploration of toxicity (confirmation of safety) is rarely an issue in the area of palliative care, when necessary, safety assessment is an objective of an exploratory study.

At the exploratory stage, assessment of study feasibility is important. "Feasible" means that the assumed patients exist and can be enrolled in the study (patient accrual), and that the study can be conducted at the study center(s) without difficulties (study operation) and can be completed by the patients (study completion). Considering that many clinical studies in palliative care fail to enroll the required number of patients or fail to be completed, the feasibility of a planned confirmatory study should be ascertained before it is performed.

In addition, appropriateness of the endpoints (including the timing and methods for endpoint data collection) selected for the confirmatory study should be assessed. Endpoints that show meaningful changes, and the appropriate timing, personnel, and methods for endpoint data collection should be determined. Typically, an exploratory study will evaluate more endpoints at more time points than a subsequent confirmatory study.

Preferably the exploratory study should use the same design as the confirmatory study (usually a randomized study), in part for the purpose of feasibility assessment. If such a study is not practical, a single-arm study or observational study with adequate analyses may be alternatively used where appropriate. When evidence on a dosage regimen is extrapolated from a study conducted overseas and when the dosage regimen is markedly different from that in Japanese practice, a single-arm feasibility study should be conducted.

5.3.2.2.2 Exploratory randomized studies

In the area of palliative care, there may be multiple treatment approaches available for a certain condition, and there is no need to select only one. In such instances, a randomized study may be conducted to determine whether there is a clinically meaningful difference in the profiles of efficacy or adverse reactions between different treatments. For example, Drug A and Drug B are available for the treatment of a certain condition and both drugs have been used, but Drug A is speculated to be more likely to cause nausea and Drug B is speculated to be more likely to cause insomnia. If the differences are shown to be clinically meaningful following hypothesis testing, Drug B would be the

most appropriate choice for patients who should avoid nausea and Drug A for patients who should avoid insomnia. As stated above, obtained data may complement existing information to aid decision making by patients or clarify future research themes.

5.3.2.2.3 Single-arm studies and observational studies

When a randomized study is not practical for certain reasons (such as when a given treatment appears to be the only option for patients with extreme pain or discomfort), or when a randomized study can be planned but its feasibility is very unlikely (such as a placebo-controlled study in imminently dying patients), estimation of the effects in a single-arm study or observational study without a subsequent confirmatory study may be appropriate.

6. Endpoints and outcome measures

This section describes concepts related to endpoints in the planning of studies in supportive care and palliative care. Although there are important points that apply to both supportive care and palliative care, this section describes the points for supportive care and palliative care under separate headings.

6.1 Endpoints and outcome measures in supportive care research

The points described below should be noted in light of the nature of supportive care.

6.1.1 Primary endpoints

6.1.1.1 Supportive care for acute toxicity affecting the outcome of cancer treatment

Given that supportive care aims to prevent or reduce the occurrence of adverse events in cancer treatment, it should ideally maximize the potential of cancer treatment and improve the treatment results. From this perspective, the primary endpoint may be survival-related outcomes such as overall survival. In reality, however, cancer treatment is the main factor affecting the treatment results and thus outcomes such as overall survival and relapse-free survival may be inappropriate efficacy endpoints for supportive care.

Even when supportive care is anticipated to improve the cancer treatment results, the primary endpoint should be the cancer treatment completion rate (a surrogate endpoint of cancer treatment) rather than survival. This is practical for shortening the study duration. An example is provided below.

Example: For supportive care for radiotherapy toxicity, the cancer treatment completion rate can be selected as the primary endpoint.

When the results show that patients who completed radiotherapy within 6 weeks have significantly

longer survival than patients who did not complete radiotherapy, acute adverse events are speculated to be responsible for half of the patients not completing radiotherapy.

→ Hypothesis: Introduction of a new supportive care will reduce the incidence of acute adverse events.

→ If the incidence of acute adverse events decreases, the cancer treatment completion rate will increase.

→ If the cancer treatment completion rate increases, this may contribute to better treatment results.

→ The treatment completion rate (a surrogate for survival) is chosen as the endpoint.

6.1.1.2 Supportive care for acute adverse events or late adverse events in cancer treatment affecting QOL

For supportive care that aims to prevent or reduce adverse events interfering with activities of daily living after the completion of treatment, endpoints unrelated to survival may be employed. However, such endpoints must be comparative, objective (accepted even by non-specialists), and reliable.

6.1.2 Other endpoints

6.1.2.1 Assurance of no interference with the effects of cancer treatment

In supportive care that aims to reduce adverse events, it is often necessary to consider the possibility that the reduction in adverse events could be due to a decreased effect of the cancer treatment. When supportive care itself may have a negative effect on cancer treatment, survival-related outcomes should be selected as co-primary endpoints.

Co-primary endpoints are multiple endpoints that must be achieved simultaneously. For example, when the incidence of grade 3 adverse events and overall survival (OS) are employed as co-primary endpoints, the new supportive care is considered effective only when the incidence of grade 3 adverse events is significantly decreased and OS is maintained above a certain level.

Examples of clinical studies that should use co-primary endpoints include a study on the effectiveness of a radioprotective agent (amifostine) for reducing adverse events in radiotherapy, or a study on the effectiveness of pre-treatment blood transfusion or erythropoietin administration for reducing myelosuppression in anticancer drug treatment.

6.1.3 Outcome measures

For assessment of adverse events, the National Cancer Institute Common Toxicity Criteria of Adverse Events (NCI-CTCAE)[23] or other widely used internationally measures should selected. Given that assessment items vary across different areas of supportive care, selection of individual scales will be described in a separate guideline.

6.2 Endpoints and outcome measures in palliative care research

6.2.1 Selection of endpoints

In clinical trials in palliative care that aim to reduce patients' symptoms, the endpoint should be patients' symptoms. Examples of endpoints include severity of pain for analgesic medications and severity of nausea/vomiting for antiemetic medications.

The most appropriate measure of symptoms is often unclear. For example, endpoints for pain can include the worst pain or average pain in 24 hours. [24] Endpoints for nausea/vomiting can include the worst nausea in 24 hours and number of vomiting episodes. As described above, even when the symptoms being treated by palliative care are clear, multiple methods are often available for their assessment. Although determination of the most important endpoints for specific types of symptoms is needed in the future, there are currently no international standardized endpoints for a large number of symptoms.

Thus, it is currently reasonable to employ a primary endpoint that is considered most important for patients and secondary endpoints that are considered relatively less important, with careful reference to prior studies. When multiple endpoints are similarly important, they should be selected as co-primary endpoints.[25]

6.2.2 Difference between primary endpoints and secondary endpoints

When multiple primary endpoints are employed but the results are inconsistent across these endpoints, interpretation of the results becomes an issue. Thus, important primary endpoints should be determined, and handling of inconsistent results should be predefined.

When the results are inconsistent between the primary endpoints and secondary endpoints, the study treatment may be regarded as being partially effective for patients. For example, if a study showed no change in the percentage of patients who did not vomit but showed a decrease in the severity of nausea or the number of vomiting episodes, the results may be interpreted as showing that the treatment is partially effective, and the treatment may be used in clinical practice if it has minor risks and if no alternative treatments are available. However, researchers should understand that the

results of secondary endpoints are not equivalent to those of primary endpoints, and should preferably conduct another clinical study to confirm the drug's efficacy using the same primary endpoint in the future.

6.2.3 Clinically meaningful difference (minimal important difference: MID) in reduction of symptoms

Clinical studies should be designed to detect the minimal important difference (MID); i.e., the smallest reduction in symptoms that is felt by individual patients as meaningful.

For outcome measures that are usually handled as continuous variables (e.g., QOL scale), evaluation based on the difference in mean scores is appropriate. However, for easier interpretation, the difference in the efficacy rate should be considered as an endpoint (for example, when the primary endpoint is "mean NRS score", a secondary endpoint can be "percentage of patients with a decrease in the NRS score of at least 2").

Currently, the MID has not been established for most types of PRO instruments. Thus, as a sensitivity analysis*, calculation of the efficacy rate using a different cutoff value is recommended. For example, when the primary endpoint is "the difference in the percentage of patients with a 33% reduction in worst pain", secondary endpoints should include "the percentage of patients with a 25% reduction in worst pain" and/or "the percentage of patients with a 50% reduction in worst pain".

6.2.4 Outcome scales

Symptom scales should be translated and validated in the user's language. Symptom assessment scales available in Japanese include the widely used Edmonton Symptom Assessment Schedule (ESAS)[26] and Brief Pain Inventory (BPI)[27].

7. Handling of deaths of subjects

7.1 Background

Appropriate assessment of serious adverse events (SAEs), which can occur frequently during clinical studies especially in palliative care, is important for both of the safety and efficiency of studies. On the premise that the occurrence of SAEs is rare, any SAE at a study site should be promptly reported in detail to the ethics committee and independent data monitoring committee.[28]

However, in clinical studies in supportive care or palliative care, particularly palliative care, the occurrence of many SAEs (including deaths) is expected because a subject's condition can worsen

due to worsening of the primary disease during the course of a study. Frequent reporting and assessment of SAEs regarding deaths due to clinical deterioration that are clearly unrelated to any study treatment can therefore not only affect the conduct of the clinical study, but may also lead to overlooking truly important SAEs.

7.2 Management of safety information

Most importantly, safety information should be appropriately managed according to applicable regulatory requirements and research design. In addition, for a study in which deaths due to deterioration of the primary disease that are unrelated to any study treatment are anticipated, the study protocol must stipulate that deaths due to deterioration in the study are not subject to expedited reporting. The specific conditions of adverse events that are not subject to expedited reporting should be determined for each study and clearly stated in the protocol. Other SAEs that are subject to expedited reporting should be reported within the designated timeframe.

When a protocol stipulates that deaths due to deterioration of the primary disease that are unrelated to any study treatment do not require expedited reporting, the following requirements must be fulfilled by the responsible researcher of the study site:

- Assessment of whether the death was expected
- Confirmation that the death was due to deterioration of the primary disease and was unrelated to any study treatment
- Confirmation using clinical documents such as medical charts

7.3 Study designs

The study design should be carefully considered, and should include a sample size calculation that takes into account subject dropouts. Additionally, in the analyses, analytical methods that minimize the effects of subject dropouts on the conclusions (i.e., are robust to missingness), or those that account for reasons for dropouts (i.e., reasons for missing data) should be used. Handling of deaths in particular requires a different approach to that used for ordinary missing data analysis, and requires caution when selecting the analysis population and other methodological issues. Specifically, unlike standard missing data analyses which attempt to estimate the treatment effect that would have been observed if (contrary to fact) all subjects had continued to be observed until the end of the study, missing data for censored cases due to death cannot exist. Thus, the target population for estimation should be limited to, for example, “patients in whom death will not occur (i.e., those who survive through the study)” (called the “principal stratum strategy”).[(29)]

8. Operational structure and quality management

8.1 Basic principals

The target quality level differs across studies, including clinical studies in supportive care and palliative care. Study costs, time, and feasibility should be considered according to applicable regulatory requirements and the research design. In addition to the protection of subjects and quality assurance, efficiency and cost-effectiveness should be considered, and a research quality management system (including the operational structure) should be established. Specifically, the research operations office and clinical research support organizations of a data center or a data manager, biostatistician, and clinical research associate (as needed) are ideal personnel. Given that research methods can differ depending on the research organization, a flexible and feasible system is needed.

In addition, rules regarding authorship, which should reflect the degree of contribution by those involved in planning and conducting the study, should be established with a consensus among research collaborators before the start of the study because supportive/palliative care studies typically involve multiple departments, multiple specialists, and multiple study sites.

8.2 Quality management of research and establishment of a quality management system (QMS)[30]

Quality management refers to a series of processes for ensuring that the quality of products or services in economical production meets the customer's requirements. The main activities of quality management are quality control and quality assurance, as described below.[31]

A customer is an organization or person that receives a product or service. In the context of clinical research, the end customer is the patient but the direct customer can vary depending on the research stage or circumstances, with potential customers including researchers, pharmaceutical companies, or regulatory authorities.

Quality refers to the degree to which the overall inherent characteristics of a product or service satisfy the requirements. In the context of clinical research, customer satisfaction is a measure of quality as assessed by the customer, where the required level is fulfillment of the GCP and other regulatory standards.

8.3 Quality control (QC) of research

Quality control refers to the activities associated with setting and implementing procedures to ensure the validity and reliability of research data and processes in the planning stage, and checking for proper conduct of research, with data correction and process modification performed as needed in

the event of improper conduct of research. Important activities of quality control are described below. To appropriately improve these quality control activities, a quality control system should be established before initiating the research. Specifically, the procedures should be standardized (e.g., preparation of standard operating procedures [SOPs], manuals) and appropriately conducted. To improve the quality of research, procedures should be checked and feedback should be provided. In the case of improper conduct of procedures, individual units of each procedure should undergo process correction/improvement with verification. Continuous quality improvement (spiral-up) activities using the plan-do-check-act (PDCA) cycle are important.

8.3.1 Research protocol preparation

Each research protocol should be complete and ensure scientific and ethical validity of the research. Useful methods for this include protocol standardization using a protocol manual, cooperation between the research operation office and the data center, and a review by a protocol review committee or a third party.

8.3.2 Enrolment and randomization

To prevent enrolment of ineligible subjects and erroneous enrollment and to ensure blinding of the randomized results, randomization by a biostatistician using appropriate methods and procedures is necessary. Useful methods for this include establishment of a central enrollment center (a data center independent of the researchers) and a web-based enrolment system.

8.3.3 Data measurement and assessments

Measurement methods are needed to avoid biases in the assessment and reporting by attending physicians and to minimize inter-center and inter-researcher variability. Useful methods for this include masking of the medication or treatment method, use of standardized endpoints, use of a unified measurement method, training, pre-study education, pre-study practice, central measurements, central assessment by a third party, central data management and central monitoring (by the data center). Adequate strategies should be put in place to avoid missing data for the primary endpoint. If missing data occurs, information should be collected regarding the reason and causative data/process to aid review in the analysis.

8.3.4 Research progress management and monitoring

Appropriate implementation of the study at each site according to the protocol and applicable written procedures should be verified. Protection of study participants' rights and safety, proper reporting of data, and proper recording of activities should be checked. Where necessary, revision of study-related documents and improvement of study processes should be considered, or improvements at a

site should be requested. Methods to enable this include self-check by researchers, project management and central monitoring by the data center (or research secretariat), on-site monitoring, and establishment of an independent data monitoring committee (to ensure safety and objectivity of assessments). Feedback to the researchers should be provided according to the risk level; i.e., network level (risk applies to all studies), protocol level (risk is study specific and applies to all sites in the study), or site/researcher level (risk is site/researcher specific).

8.3.5 Data management

It is important to ensure that the data are accurate and complete, and that the level of data quality is sufficiently high to allow fair and scientific assessments. Useful methods for this include establishing a data center independent of the researchers and use of a validated data management system. Data management is a method used to efficiently collect and manage accurate, precise, high-quality data to enable fair data assessments that lead to accurate and valid conclusions. Data management has an important role in all processes of a clinical study, including preparation of the study protocol, preparation of case report forms, data collection, data verification, inquiries to researchers, study progress management, data entry, data cleaning, and determination of analysis data sets. Notably, the quality of a clinical study depends on the data, and the role of data management is beyond simple data collection, entry, and check.

8.3.6 Data collection process management at each study site

For data quality management by site (researcher), data collection should be conducted in accordance with the principles of ALCOA (i.e., attributable, legible, contemporaneous, original, and accurate). It is also important to clarify the operational structure of the site, including the roles and responsibilities of researchers and collaborators and information sharing procedures, before initiating the study.

8.3.7 Statistical analysis

The analysis process must be validated. A statistical analysis plan (that considers the analysis sets and data handling) should be prepared before initiating the study, and a validated statistical analysis package (software) should be used. Where possible, double programming and other relevant methods should be used to ensure reproducibility of the results.

8.4 Research quality assurance (QA)

Research quality control activities are initiated by researchers, and thus may not meet customer needs. Quality assurance refers to the activities performed to ensure that the quality of the study is above the level required by the customer. For quality assurance, a third person should check the

study records (proofs) generated during the course of the study to determine whether the study was properly conducted based on the data and process management. Quality assurance involves reviewing the quality of the entire study; i.e., all study processes including evaluations, assessments, and results, to ensure proper conduct of the study. Auditing is a core activity in quality assurance.

References

1. Hui D. Definition of supportive care: does the semantic matter? Curr Opin Oncol. 2014;26(4):372-9.
2. U.S. National Institutes of Health. NCI Dictionary of Cancer Terms [Available from: https://www.cancer.gov/publications/dictionaries/cancer-terms.]
3. World Health Organization. WHO Definition of Palliative Care [Available from: http://www.who.int/cancer/palliative/definition/en/.]
4. Randomized, double-blind, placebo-controlled study to assess the efficacy and toxicity of subcutaneous ketamine in the management of cancer pain. Hardy J, Quinn S, Fazekas B, Plummer J, Eckermann S, Agar M, Spruyt O, Rowett D, Currow DC. J Clin Oncol. 2012 Oct 10;30(29):3611-7. doi: 10.1200/JCO.2012.42.1081. Epub 2012 Sep 10.
5. *Caraceni A. J Clin Oncol. 2004;22:2909-17.*
6. Fentanyl Sublingual Tablets Versus Subcutaneous Morphine for the Management of Severe Cancer Pain Episodes in Patients Receiving Opioid Treatment: A Double-Blind, Randomized, Noninferiority Trial. Zecca E, Brunelli C, Centurioni F, Manzoni A, Pigni A, Caraceni A. J Clin Oncol. 2017 Mar;35(7):759-765. doi: 10.1200/JCO.2016.69.9504. Epub 2017 Jan 23.
7. Treede RD, Jensen TS, Campbell JN, Cruccu G, Dostrovsky JO, Griffin JW, et al. Neuropathic pain: redefinition and a grading system for clinical and research purposes. Neurology. 2008;70(18):1630-5.
8. American Psychiatric Association. Diagnostic and Statistical Manual of Mental Disorders. 5th edition: Amer Psychiatric Pub Inc; 2013.
9. Hui D. Prognostication of Survival in Patients With Advanced Cancer: Predicting the Unpredictable? Cancer Control. 2015;22(4):489-97.
10. Oken MM, Creech RH, Tormey DC, Horton J, Davis TE, McFadden ET, et al. Toxicity and response criteria of the Eastern Cooperative Oncology Group. Am J Clin Oncol. 1982;5(6):649-55.
11. Karnofsky DA, Burchenal JH. The clinical evaluation of chemotherapeutic agents in cancer, in Macleod CM (ed): Evaluation of Chemotherapeutic Agents. New York: Columbia University Press; 1949.
12. Maltoni M, Nanni O, Pirovano M, Scarpi E, Indelli M, Martini C, et al. Successful validation of the palliative prognostic score in terminally ill cancer patients. Italian Multicenter Study Group on Palliative Care. Journal of pain and symptom management. 1999;17(4):240-7.

13. Morita T, Tsunoda J, Inoue S, Chihara S. The Palliative Prognostic Index: a scoring system for survival prediction of terminally ill cancer patients. Supportive care in cancer : official journal of the Multinational Association of Supportive Care in Cancer. 1999;7(3):128-33.

14. Gwilliam B, Keeley V, Todd C, Gittins M, Roberts C, Kelly L, et al. Development of prognosis in palliative care study (PiPS) predictor models to improve prognostication in advanced cancer: prospective cohort study. BMJ. 2011;343:d4920.
15. Masso M, Allingham SF, Banfield M, Johnson CE, Pidgeon T, Yates P, et al. Palliative Care Phase: inter-rater reliability and acceptability in a national study. Palliat Med. 2015;29(1):22-30.
16. Folstein MF, Folstein SE, McHugh PR. "Mini-mental state". Journal of Psychiatric Research. 1975;12(3):189-98.
17. Loudon K, Treweek S, Sullivan F, Donnan P, Thorpe KE, Zwarenstein M. The PRECIS-2 tool: designing trials that are fit for purpose. BMJ. 2015;350:h2147. PMID: 25956159.
18. Ross C. Brownson, Graham A. Colditz and Enola K. Proctor. Dissemination and Implementation Research in Health: Translating Science to Practice. Oxford University Press.
19. Fentanyl Sublingual Tablets Versus Subcutaneous Morphine for the Management of Severe Cancer Pain Episodes in Patients Receiving Opioid Treatment: A Double-Blind, Randomized, Noninferiority Trial. Zecca E, Brunelli C, Centurioni F, Manzoni A, Pigni A, Caraceni A. J Clin Oncol. 2017 Mar;35(7):759-765. doi: 10.1200/JCO.2016.69.9504. Epub 2017 Jan 23.
20. Effect of continuous deep sedation on survival in patients with advanced cancer (J-Proval): a propensity score-weighted analysis of a prospective cohort study. Maeda I, Morita T, Yamaguchi T, Inoue S, Ikenaga M, Matsumoto Y, Sekine R, Yamaguchi T, Hirohashi T, Tajima T, Tatara R, Watanabe H, Otani H, Takigawa C, Matsuda Y, Nagaoka H, Mori M, Tei Y, Kikuchi A, Baba M, Kinoshita H. Lancet Oncol. 2016 Jan;17(1):115-22. doi: 10.1016/S1470-2045(15)00401-5. Epub 2015 Nov 29.
21. Parenteral hydration in patients with advanced cancer: a multicenter, double-blind, placebo-controlled randomized trial. Bruera E, Hui D, Dalal S, Torres-Vigil I, Trumble J, Roosth J, Krauter S, Strickland C, Unger K, Palmer JL, Allo J, Frisbee-Hume S, Tarleton K. Ann Oncol. 2005 Apr;16(4):640-7. Epub 2005 Jan 31.
22. Association between hydration volume and symptoms in terminally ill cancer patients with abdominal malignancies. Morita T1, Hyodo I, Yoshimi T, Ikenaga M, Tamura Y, Yoshizawa A, Shimada A, Akechi T, Miyashita M, Adachi I; Japan Palliative Oncology Study Group. J Clin Oncol. 2013 Jan 1;31(1):111-8. doi: 10.1200/JCO.2012.44.6518. Epub 2012 Nov 19.
23. U.S. NCI Division of Cancer Treatment and Diagnosis. Common Terminology Criteria for Adverse Events (CTCAE) Version 4.0 2009 [updated May 28. Available from: https://ctep.cancer.gov/protocoldevelopment/electronic_applications/ctc.htm.]
24. U.S. Food and Drug Administration. Guidance for industry analgesic indications: developing drug and biological products. 2014.
25. U.S. Food and Drug Administration. Guidance for industry: patient-reported outcome

measures: use in medical product development to support labeling claims. Federal Register2009. p. 65132-3.

26. Bruera E, Kuehn N, Miller MJ, Selmser P, Macmillan K. The Edmonton Symptom Assessment System (ESAS): a simple method for the assessment of palliative care patients. J Palliat Care. 1991;7(2):6-9.

27. Cleeland CS1 RK. Pain assessment: global use of the Brief Pain Inventory. Ann Acad Med Singapore. 1994;23(2):129-38.

28. Ministry of Education, Culture, Sports, Science and Technology and Ministry of Health, Labour and Welfare. Ethical Guidelines for Medical and Health Research Involving Human Subjects.

29. Addendum to Statistical Principles for Clinical Trials on Choosing Appropriate Estimands and Defining Sensitivity Analyses in Clinical Trials (https://www.pmda.go.jp/int-activities/int-harmony/ich/0031.html) 2)

30. International Council for Harmonisation of Technical Requirements for Pharmaceuticals for Human Use. INTEGRATED ADDENDUM TO ICH E6(R1): GUIDELINE FOR GOOD CLINICAL PRACTICE E6(R2). 2016.

31. INTEGRATED ADDENDUM TO ICH E6(R1): GUIDELINE FOR GOOD CLINICAL PRACTICE E6(R2) (https://www.pmda.go.jp/int-activities/int-harmony/ich/0028.html)
